# Decoding the colorectal cancer ecosystem emphasizes the cooperative role of cancer cells, TAMs and CAFsin tumor progression

**DOI:** 10.1186/s12967-022-03661-8

**Published:** 2022-10-08

**Authors:** Rongfang Shen, Ping Li, Botao Zhang, Lin Feng, Shujun Cheng

**Affiliations:** 1 State Key Laboratory of Molecular Oncology, Department of Etiology and Carcinogenesis, National Cancer Center/National Clinical Research Center for Cancer/Cancer Hospital, Chinese Academy of Medical Sciences and Peking Union Medical College, NO. 17 Panjiayuannanli, Chaoyang District, Beijing, 100021 China; 2 Medical Oncology Department, Pediatric Oncology Center, Beijing Children’s Hospital, Capital Medical University, National Center for Children’s Health, Beijing Key Laboratory of Pediatric Hematology Oncology, Key Laboratory of Major Diseases in Children, Ministry of Education, Beijing, 100045 China; 3 Department of Neuro-Oncology, Cancer Center, Beijing Tiantan Hospital, Capital Medical University, Beijing, China

**Keywords:** Colorectal cancer, scRNA-seq, Tumor heterogeneity, Epithelium-microenvironment communication, Tumor-associated macrophages, Cancer-associated fibroblasts

## Abstract

**Background:**

Single-cell transcription data provided unprecedented molecular information, enabling us to directly encode the ecosystem of colorectal cancer (CRC). Characterization of the diversity of epithelial cells and how they cooperate with tumor microenvironment cells (TME) to endow CRC with aggressive characteristics at single-cell resolution is critical for the understanding of tumor progression mechanism.

**Methods:**

In this study, we comprehensively analyzed the single-cell transcription data, bulk-RNA sequencing data and pathological tissue data. In detail, cellular heterogeneity of TME and epithelial cells were analyzed by unsupervised classification and consensus nonnegative matrix factorization analysis, respectively. Functional status of epithelial clusters was annotated by CancerSEA and its crosstalk with TME cells was investigated using CellPhoneDB and correlation analysis. Findings from single-cell transcription data were further validated in bulk-RNA sequencing data and pathological tissue data.

**Results:**

A distinct cellular composition was observed between tumor and normal tissues, and tumors exhibited immunosuppressive phenotypes. Regarding epithelial cells, we identified one highly invasiveQuery cluster, C4, that correlated closely with tumor-associated macrophages (TAMs) and cancer-associated fibroblasts (CAFs). Further analysis emphasized the TAMs subclass TAM1 and CAFs subclass S5 are closely related with C4.

**Conclusions:**

In summary, our study elaborates on the cellular heterogeneity of CRC, revealing that TAMs and CAFs were critical for crosstalk network epithelial cells and TME cells. This in-depth understanding of cancer cell-TME network provided theoretical basis for the development of new drugs targeting this sophisticated network in CRC.

**Supplementary Information:**

The online version contains supplementary material available at 10.1186/s12967-022-03661-8.

## Novelty and impact

Colorectal cancer cells communicate closely with the surrounding environment. However, which cell plays the key role in this communication network remains unclear. By comprehensively analyzing single-cell transcription data, the authors found 7 functional heterogenous cancer clusters and discovered one highly invasive cluster: C4. C4 was tightly connected with TAMs and CAFs, and the relationship between C4 and TAMs was validated. This study provides comprehensive insights into the heterogeneity of cancer cells and reveals that TAMs play a crucial role in the cancer cell-tumor environment network.

## Background

CRC is one of the most prevalent tumors, with a high mortality rate [1]. Routine therapies include surgery, chemotherapy and radiotherapy. However, satisfactory clinical outcomes have not been achieved due to tremendous intratumoral heterogeneity [2].Molecular classifications of CRC have revealed immunosuppressive tumor environments, which are closely linked to adverse survival, in patients with high stromal infiltration [3–5].Recently, breakthrough immunotherapies emphasizing cytotoxic T cell enhancement have made great progress in prolonging the survival of patients with lung cancer, [6] melanoma [7] and bladder cancer [8].Patients with microsatellite instability or a high Immunoscore, as defined by T-cell infiltration and distribution patterns, [9, 10] are more likely to obtain survival benefit from immune checkpoint blockade treatment. Nevertheless, these patients constitute only a minor proportion of those with CRC, suggesting the need to investigate new therapeutic strategies targeting other cells, such as myeloid cells [11] or stromal-lineage cells, [12] or the entire cell‒cell communication network. Overall, a comprehensive understanding of the CRC ecosystem is a prerequisite for discovering effective therapeutic strategies.

The heterogeneity of tumor cells and tumor microenvironment (TME) cells plays a vital role in shaping cellular biological behaviors [13].Supporting cells in the TME have critical roles in maintaining tissue homeostasis in health [14] or promoting cancer progression in the presence of tumors [15].The complex interplay between the epithelium and microenvironment is of pivotal importance for oncogenesis and tumor progression [16, 17].The advent of single-cell RNA sequencing (scRNA-seq) technology has provided unprecedented molecular information and enabled us to systematically decipher the complexity of tumor biology [18–20].Zemin Zhang and colleagues [21] described the T-cell atlas of CRC and further explored new treatment strategies targeting myeloid cells. [11] Woong-Yang Park et al. [22] clarified the influence of cancer cell programs on the immune landscape of CRC. These studies have deepened our understanding of the cellular and transcriptional features of CRC, especially its surrounding tumor environment. However, the cellular heterogeneity of the colorectal epithelium, its complex interplay with environmental cells and how they orchestrate the initiation of tumorigenesis and the promotion of tumor progression are still poorly understood.

In this study, we aimed to dissect the cellular and transcriptional diversity of the epithelium compartment (EC) and the microenvironment compartment (MC) in CRC and tumor-adjacent tissue samples by integrating scRNA-seq data. Herein, we first describe the cellular heterogeneity of the EC and MC in two scRNA-seq datasets, the Samsung Medical Center (SMC) and Katholieke Universiteit Leuven (KUL3) cohorts, and compare their cellular compositions between tumor and adjacent tissue samples. Further analysis underscores the critical role of TAMs and CAFs in influencing patient survival and controlling the communication network between the EC and MC. For EC analysis, we identified one cancer cell cluster, C4, which features an aggressive phenotype. Tumor samples with the C4 phenotype tend to have more infiltrating immunosuppressive immune and stromal cells, such as TAMs and CAFs. Further analysis of these TAMs and CAFs revealed one TAM subclass (TAM1) and one CAF subclass (S5) that are closely linked with the aggressive epithelial phenotype. The adverse role of TAM1 and the correlation between TAM1 and C4 were validated in vitro via multiplex immunohistochemistry (IHC) analysis of 220 CRC patients.

## Materials and methods

### Public data access and processing

We systematically search colorectal cancer scRNA-seq data publicly available. Datasets with immune cell sorting (magnetic-activated cell sorting or fluorescence-activated cell sorting) [11, 21]were excluded due to the lack of epithelial cells and distorted cell proportions. Here, we included 2 cohorts generated by 10 × Genomics single-cell 3’ sequencing platform: SMC and KUL3 cohort [22]. Patients from both cohorts did not receive any prior treatment before surgery, covering stage I-IV with diverse tumor locations. SMC cohort contained 10 normal tissues and 23 tumor samples, and paired tumor core, border and normal tissue from 6 colorectal cancer patients were included in KUL3 cohort. Patients’ basic clinical features were summarized in Additional file 1: Table S1 and detailed information can be found in the source study [22] (https://static-content.springer.com/esm/art%3A10.1038%2Fs41588-020-0636-z/MediaObjects/41588_2020_636_MOESM3_ESM.xlsx). To validate the findings derived from scRNA-seq data in a larger population, we included the TCGA-COADREAD cohort, for which primary tumors without survival information were excluded. Filtered unique molecular identifier (UMI) count matrices of the SMC cohort and KUL3 cohort deposited in the Gene Expression Omnibus (GEO, https://www.ncbi.nlm.nih.gov/geo/, RRID:SCR_005012) database under accession numbers GSE132465 and GSE144735 were downloaded. CRC transcriptome profiling data from TCGA were retrieved using the R package “TCGAbiolinks” [23] in the HTSeq-FPKM workflow. Fragments per kilobase million (FPKM) values were transformed to transcripts per million (TPM) values, and the log2(TPM + 1) method was used to normalize expression levels in subsequent analysis. Survival data for TCGA-COADREAD were downloaded from the UCSC Xena browser (https://xenabrowser.net).

### Clinical samples for multiplex IHC and IHC

To further validated in vitro, we purchased colorectal cancer tissue microarray chips from Shanghai Outdo Biotech Company (Shanghai, China). Rectal tumors (tissue array 1, n = 90, array ID: HRec-Ade180Sur-05) and colon tumors (tissue array 2, n = 100, array ID: HCol-A180-Su10) with overall survival information were selected for further validation. Colon tumors (tissue array 3, n = 30, array ID: HCol-A030PG-06) without survival information were also included to evaluate the relationship between TAM1 and C4. Clinical features including tumor types, gender, age, tumor location, tumor size, TNM stage and tumor grade are summarized in Table 1. Samples without sufficient tissue remaining were excluded. Two pathologists further confirmed the diagnosis of CRC.

**Table 1:** Clinical characteristics of patients enrolled in the validation cohort

		Tissue Array 1 (n = 90)	Tissue Array 2 (n = 100)	Tissue Array 3 (n = 30)
Cancer Type (%)	COAD	0 (0.0)	100 (100.0)	30 (100.0)
	READ	90 (100.0)	0 (0.0)	0 (0.0)
Sex (%)	Female	42 (46.7)	41 (41.4)	13 (43.3)
	Male	48 (53.3)	58 (58.6)	17 (56.7)
Age (Mean (SD))		65.86 (11.64)	67.51 (10.74)	56.28 (17.50)
Location (%)	Left	90 (100.0)	42 (42.0)	10 (33.3)
	Right	0 (0.0)	50 (50.0)	16 (53.3)
	Unknown	0 (0.0)	8 (8.0)	4 (13.3)
Survival (%)	OS	90 (100.0)	100 (100.0)	0 (0.0)
	Unknown	0 (0.0)	0 (0.0)	30 (100.0)
Tumor Size (%)	> = 5	45 (50.0)	63 (63.6)	21 (70.0)
	< 5	45 (50.0)	36 (36.4)	8 (26.7)
	Unknown	0 (0.0)	0 (0.0)	1 (3.3)
T Stage (%)	T1	2 (2.2)	0 (0.0)	0 (0.0)
	T2	10 (11.2)	4 (4.0)	0 (0.0)
	T3	76 (85.4)	64 (64.0)	0 (0.0)
	T4	1 (1.1)	32 (32.0)	0 (0.0)
	Unknown	0 (0.0)	0 (0.0)	30 (100.0)
N Stage (%)	N0	54 (60.0)	52 (52.0)	28 (93.3)
	N1	24 (26.7)	36 (36.0)	0 (0.0)
	N2	12 (13.3)	12 (12.0)	0 (0.0)
	Unknown	0 (0.0)	0 (0.0)	2 (6.7)
M Stage (%)	M0	89 (98.9)	95 (95.0)	30 (100.0)
	M1	1 (1.1)	5 (5.0)	0 (0.0)
AJCC7 Stage (%)	Stage I	12 (13.3)	4 (4.0)	0 (0.0)
	Stage II	41 (45.6)	47 (47.0)	0 (0.0)
	Stage III	36 (40.0)	44 (44.0)	0 (0.0)
	Stage IV	1 (1.1)	5 (5.0)	0 (0.0)
	Unknow	0 (0.0)	0 (0.0)	30 (100.0)
Tumor Grade (%)	I	3 (3.3)	0 (0.0)	2 (6.7)
	I-II	13 (14.4)	14 (14.0)	4 (13.3)
	I-III	0 (0.0)	3 (3.0)	0 (0.0)
	II	62 (68.9)	56 (56.0)	11 (36.7)
	II-III	6 (6.7)	18 (18.0)	4 (13.3)
	III	6 (6.7)	9 (9.0)	9 (30.0)
In vitro Validation	IHC	Yes	No	Yes
	Multiplex IHC	M Panel	M & F Panels	M & F Panels

### Unsupervised classification and cell type annotation

Filtered UMI count matrices provided by the Woong-Yang Park group were downloaded and transformed into Seurat objects. Cells retained in the matrices all satisfied the following criteria: more than 1000 UMIs, expressed between 200 and 6000 genes, and fewer than 20% mitochondrion-derived UMIs. The R package “Seurat” (v3.1.5) was used for normalization, dimension reduction and unsupervised graph-based clustering. Principal component analysis (PCA) was performed using 5000 variable features, and the number of components used for clustering was determined based on the variance explained percentage derived from the “ElbowPlot” function. A shared nearest neighbor (SNN) graph was constructed based on the neighborhood overlap of each cell and its 20 nearest neighbors. The Louvain algorithm was implemented to find cell clusters within 0.4–0.6 resolution. Uniform manifold approximation and projection (UMAP) analysis was applied to visualize low-dimensional space scaling data. No cellularity bias or batch effect was found among subgroups. Thus, no further data integration was adopted in this study.

To assign specific cells to each cluster, we first identified cluster biomarkers through the “FindAllMarkers” function and compared these biomarkers with canonical cell markers. Specifically, T cells were annotated based on their high expression of *CD3D* and *CD3E* and lack of expression of *EPCAM* (expression of which suggests epithelial origin), B cells based on their expression of *CD79A* and *MS4A1*, plasma cells based on their expression of *SDC1* and *MZB1*, myeloid cells based on their expression of CD68, endothelial cells based on their expression of *ENG* and *VWF*, and stromal cells based on their expression of *DCN*. Seven major cell types, including epithelial cells, T cells, B cells, plasma cells, myeloid cells, endothelial cells and stromal cells, and three minor cell types, including enteric glial cells, mast cells and plasmacytoid dendritic cells (pDCs), were identified. Major cell types were reclustered into subsets using the same workflow from the first-round clustering, with the exception of 2000 highly variable features. Small cell clusters with coexpression of markers of different lineages, such as *CD3D* and *EPCAM*, were considered doublet cells, as such markers are unlikely to colocalize. We noticed that one cluster in the SMC cohort with a small cell number showed coexpression of goblet cell markers such as *SPINK4*, *AGR2*, and *REG4* and was distributed in all major cells. These cells were considered sample contamination because the origin of all cells was patient “SMC-20”, who had mucinous adenocarcinoma. In addition, cells with high expression of heat shock proteins were excluded from downstream analysis.

### Differential expression analysis and biological function enrichment analysis of epithelial cells

Pseudobulk expression profiles of epithelial cells in the SMC and KUL3 cohorts were constructed by summing the UMI counts of all tumor and normal epithelial cells in each patient, as previously described [24].Genes with a detected percentage lower than 0.25 in epithelial cells were excluded from differential expression analysis. The R package “edgeR”, [25] a differential expression analysis tool previously used for bulk RNA-seq analysis, was implemented to identify DEGs. Genes with adjusted p values less than 0.05 and absolute log2-fold changes larger than 1 were considered DEGs. The R package “clusterProfiler” [26] was used to perform biological function enrichment analysis.

### Estimating cell composition by deconvolution

Relative cell proportions in the cohort TCGA-COADREAD were estimated using CIBERSORTx, a widely acknowledged tool for performing digital cytometry [27].To counteract the high drop-out rate effect and distinct sequencing depth bias, we constructed a single-cell reference matrix by summing the UMI counts of 10 randomly chosen cells within each cell type [28].This modification largely reduced the computational memory cost and improved the robustness of reference construction. The module “create signature matrix” at the CIBERSORTx website was used to construct a CRC signature reference matrix (CRC-SRM) with the following parameters: disable quantile normalization = true, kappa = 99, q-value = 0.01, No. barcode genes = 300 to 500, min. expression = 1, replicates = 0, sampling = 0, and filter nonhematopoietic genes from signature matrix during construction = false. Counts per million-normalized transcriptome data from TCGA-COADREAD were prepared to determine cell proportions using the generated CRC-SRM file according to the instructions of the CIBERSORTx website. S-mode batch correction was performed considering that the mixture matrix and signature reference matrix were generated by different platforms. The remaining parameters were set as follows: disable quantile normalization = true, run mode (relative or absolute) = relative, and permutations = 100.

### Epithelial cell clustering

Consensus nonnegative matrix factorization (cNMF) [29] was applied to discover gene expression programs in tumor samples with an epithelial cell number larger than 100 in the SMC cohort. In total, 22 tumor samples were included. For each sample, the optimal k value (number of components) was selected considering the balance between clustering stability and error. The top 100 genes with the highest contribution to each gene program were extracted, and their relative enrichment level was inferred with the R package “AUcell” [30].Pearson correlation and hierarchical cluster analyses were employed to identify functional metaprograms. The relative enrichment score of the identified gene programs was calculated with “AUcell”. Then, we implemented hierarchical clustering to identify epithelial cell clusters. Corresponding clusters in the KUL3 cohort were identified by implementing SingleR [31].

### Epithelial cell differentiation status determination

The differentiation status of individual cells was inferred based on single-cell entropy (scEntropy). [32] Cells with high scEntropy have high plasticity, stem-like cell characteristics, and the potential to differentiate into multiple lineages.

### Cell‒cell communication analysis

Crosstalk between identified cell clusters was qualified using CellPhoneDB (RRID:SCR_017054), [33, 34] a ligand and receptor repository and statistical framework for predicting potential interactions between cell types. The cell transcriptomes of the identified cell clusters derived from the tumor samples in log-TPM normalized form were subjected to CellPhoneDB under the following parameters: subsampling-log = false, subsampling-num-cells = 10,000, counts-data = hgnc_symbol, and iterations = 100. Interactions whose p value was less than 0.05 were considered potential interaction pairs. We used Cytoscape (RRID:SCR_003032) to construct and visualize the interaction networks. A dot plot was generated to demonstrate the detailed interaction patterns. The same procedures and parameters were implemented for adjacent normal samples.

### Gene regulatory network analysis

Regulons (transcription factors and their target gene regulatory networks) were inferred by pySCENIC (RRID:SCR_017247) [30], a lightweight Python (RRID:SCR_001658) implementation of the Single-Cell rEgulatory Network Inference and Clustering (SCENIC) pipeline. Regulon activity was qualified through determination of the area under the curve (AUC) value by the “AUcell” package. The Regulon specificity score (RSS) calculated with entropy-based metrics was used to define regulon occupancy. [28] The top 5 regulons with the highest RSS of each cell subtype were considered hub regulons.

### Pseudotime trajectory analysis

To explore cellular development, differentiation and cellular fate conversions, single-cell trajectories were constructed by Monocle2 (RRID:SCR_016339), [35] which implements reversed graph embedding to identify transition branches. Differentially expressed biomarkers of individual clusters were pooled and chosen to represent dynamic changes in the differentiation process among predefined clusters. Reduced dimension graphs and pseudotime heatmaps were generated by the “plot_cell_trajectory” and “plot_pseudotime_heatmap” functions in Monocle2.

### IHC

Tissue arrays 1 and 3 were employed for IHC. FFPE slides were baked in an oven at 65 °C for 4–6 h, dewaxed using xylene, rehydrated using ethanol solutions and incubated with 3% hydrogen peroxide for 10 min. Antigen retrieval was performed by microwave treatment (MWT) with citrate buffer (pH 6.0). After blocking with a nonspecific antibody, an anti-LAMB3 antibody (Santa Cruz Biotechnology Cat# sc-133178) or anti-ERO1A antibody (Abcam Cat# ab177156) was added to the samples and incubated overnight at 4 °C. DAB was applied for staining. IHC scores ranged from 0 to 12 and were calculated by multiplying the staining intensity score (ranging from 0 to 3: 0, negative staining; 1, weak staining; 2, moderate staining; and 3, strong staining) with the score for the proportion of positive cells (ranging from 0 to 4: 0, negative; 1, < 10% positive cells in the staining area; 2, 10%-50% positive cells in the staining area; 3, 50–75% positive cells in the staining area; and 4, > 75% positive cells in the staining area).

### Multiplex IHC staining

Multicolor immunofluorescence staining was performed using a PANO IHC kit (Panovue, Beijing, China). The deparaffinization and rehydration processes were similar to those used for IHC. Antigen retrieval was performed by MWT with antigen retrieval buffer (pH 6.0 or pH 9.0). Primary antibody incubation was followed by addition of secondary antibody-HRP solution and fluorophore working solution to generate a fluorescence signal. As the fluorescence signal is generated by covalent binding and not affected by MWT, MWT was then applied to remove the detected antibody, with the fluorescence signal being conserved. The procedures including MWT, primary antibody incubation, secondary-HRP solution incubation and signal generation were repeated for the remaining antibodies. Nuclei was stained with 4′-6′-diamidino-2-phenylindole (DAPI, SIGMA-ALDRICH), and the slides were then mounted with mounting medium and coverslipped. The primary antibodies used for multiplexed IHC staining targeted the following molecules: CD68 (Cell Signaling Technology Cat# 76,437, RRID: AB_2799882), CD163 (Cell Signaling Technology Cat# 93,498, RRID: AB_2800204), S100A8 (Proteintech Cat# 66,853–1-Ig, RRID: AB_2882193), IDO1 (Abcam Cat# ab211017), VIM (Cell Signaling Technology Cat# 5741, RRID: AB_10695459), WT1 (Cell Marque Cat# 348 M-96, RRID: AB_1161125) and FAP (Abcam Cat# ab207178, RRID: AB_2864720).

### Image analysis

Images were captured at 20 × magnification (Vectra Polaris, Perkin Elmer, USA) and analyzed with inForm 2.4 software, including spectral unmixing, tissue segment, cell segment, phenotype and IHC scoring. Tissues were segmented into the EC and MC, and cells were segmented into nucleus, cytoplasm, and membrane compartments. The phenotyping algorithm was trained by assigning 30–50 cells to each phenotype. For example, cells were classified as CD68^+^, CD163^+^, IDO1^+^, S100A8^+^ and others for the macrophage biomarker panel (M panel) and as VIM^+^, FAP^+^, WT1^+^ and others for the fibroblast biomarker panel (F panel). For each molecular component, the threshold was set by summarizing the mean IHC score of tissue arrays 1–3 for each phenotype of cells at 10%. Cells were reclassified as marker-positive cells if their IHC score was higher than threshold.

### Statistical analysis

All statistical analyses were performed using R package “stats” with R version 3.6.3 (R Project for Statistical Computing, RRID:SCR_001905). Cell proportion differences were evaluated by the Wilcoxon rank-sum test (for comparisons of two groups) or Kruskal–Wallis test (for comparisons of more than two groups). The Kaplan‒Meier method was applied to estimate survival probabilities. Optimal cutoff values for survival analysis were determined by maximally selected rank statistics wrapped in the R package “survminer”. For multiple comparisons, the Benjamini and Hochberg (BH) method was used to adjust the p value; p < 0.05 was considered to indicate statistical significance.

## Results

### Decoding the cellular ecosystem at single-cell resolution

**Fig. 1:**
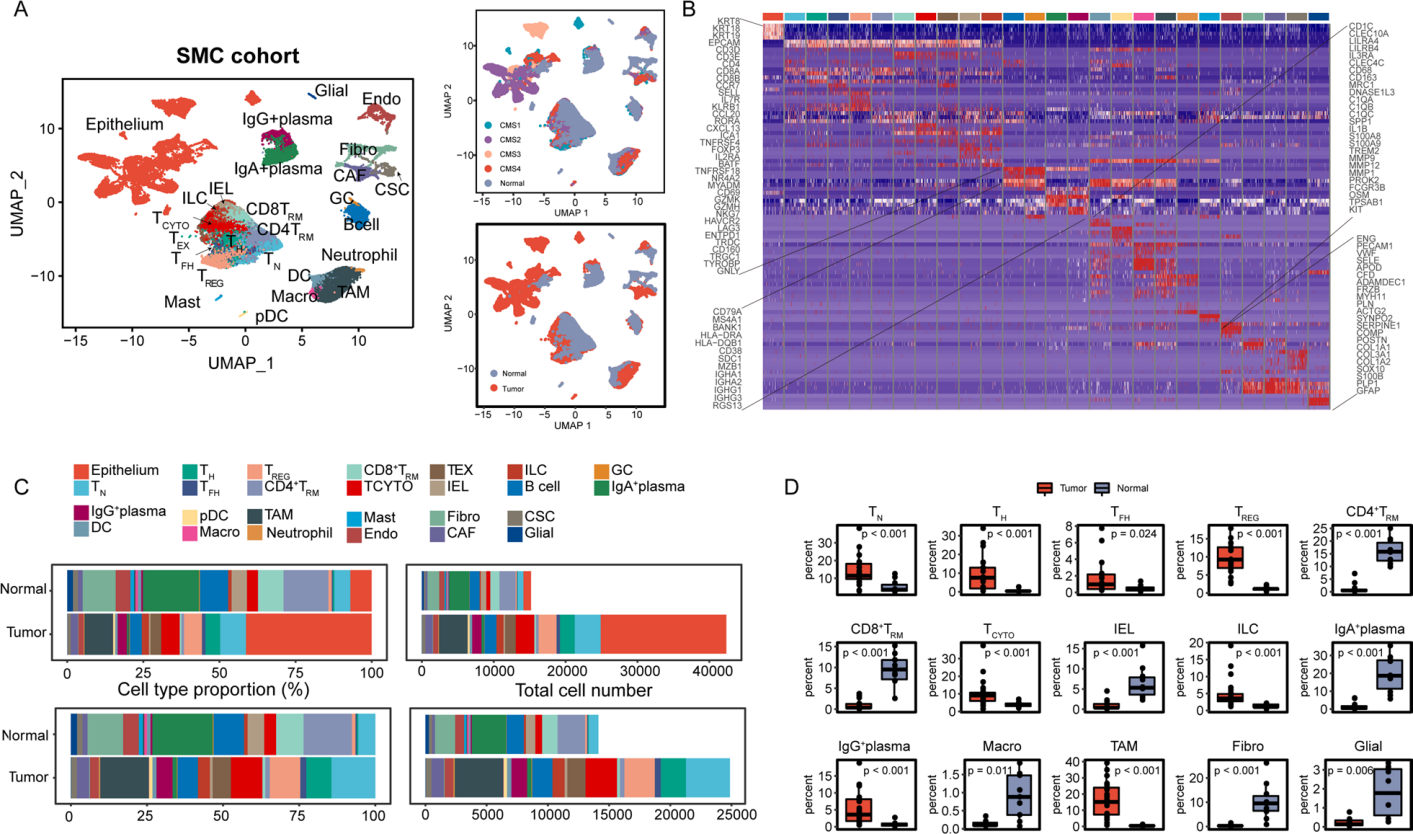
Cellular landscape of CRC in the SMC cohort. **A** UMAP plot of 57,557 cells in the SMC cohort colored by cell cluster, CMS and tissue type (tumor or normal). Each dot represents a cell and cellular cluster is annotated with text. **B**. Heatmap of representative markers of cell clusters. A total of 100 random cells in each cluster were chosen for visualization. Marker genes could also be found in Additional file 2: Table S2. The color legend is as in C. **C**. Proportions of the identified cell clusters across tumor and adjacent normal tissues with relative cell type proportions and total cell numbers. Upper: all cell clusters; bottom: immune and stromal cell clusters. **D**. Frequencies of selected cell types for tumor and adjacent normal samples. The Wilcoxon rank-sum test p value is shown

In total, 57,557 and 26,268 cells from the SMC cohort and KUL3 cohort, respectively, that passed quality control were included for further analysis (Additional file 2: Table S2). Global cell clusters, for example, epithelial cells, T cells, B cells, plasma cells, myeloid cells and stromal cells, were identified. The cellular distribution patterns between primary tumor and adjacent normal tissue samples were remarkably distinct, which may hint at the colorectal tumorigenesis mechanism (Fig. 1A, Additional file 8: Fig. S1A). Marginal differences in immune and stromal cells were observed between the tumor core and tumor border tissues (Additional file 8: Fig. S1A, B). Further classification of T cells revealed 9 subclasses based on canonical marker genes [36–38]: naïve T (T_N_), tissue-resident T (T_RM_), T helper (T_H_), T follicular helper (T_FH_), regulatory T (T_REG_), cytotoxic T (T_CYTO_), exhausted T (T_EX_) cells, intraepithelial lymphocytes (IELs, mainly γδ T cells) and innate lymphoid cells (ILCs, mainly natural killer (NK) cells). The T_N_, T_H_, T_FH_ and T_REG_ cells were mainly CD4^+^ T cells; the T_CYTO_ and T_EX_ cells and IELs were almost all CD8^+^ T cells. The lineage of T_RM_ cells was annotated as CD4T_RM_ or CD8T_RM_. A small cluster termed germinal center (GC) B cells was isolated from the B-cell cluster owing to its high expression of *RGS13* [39]*.* Plasma cells were further divided into IgA^+^ plasma and IgG^+^ plasma cells based on their different expression levels of *IGHA1*, *IGHA2*, *IGHG1* and *IGHG3* [40].Dendritic cells (DCs), pDCs, macrophages (Macro), neutrophils and TAMs were classified from myeloid cell lineages [39]. Stromal lineages were roughly categorized into endothelial cells, fibroblasts, CAFs and contractile stromal cells (CSCs) [39, 41]. Canonical cell markers of each subclass are shown in Fig. 1B and Additional file 8: Fig. S1C (Additional file 3: Table S3). TAMs and CAFs were classified according to their tumor origin and high expression of *SPP1*, *C1QB*, *IL1B*, *S100A8*, *S100A9* and type I collagens (*COL1A1* and *COL1A2*). We also identified enteric glial cells and mast cells. Enteric glial cells, mast cells, GC B cells and neutrophils were excluded from the CIBERSORTx and cell‒cell communication network analyses because of their small cell numbers.

Few epithelial cells were detected in adjacent normal tissues, possibly due to their vulnerability to damage during tissue dissociation (Fig. 1C top, Additional file 8: Fig. S1B top). The cellular composition of adjacent normal tissues mainly included T_RM_ cells, B cells, IgA^+^ plasma cells and fibroblasts. Conversely, the tumor sample cellular composition was much more heterogeneous (Fig. 1C bottom, Additional file 8: Fig. S1B bottom). Tumor tissues were enriched for T_N_, T_H_, T_FH_, T_REG_, and T_CYTO_ cells and ILCs and had fewer T_RM_ cells and IELs than adjacent normal tissues. Regarding non-T cells, higher percentages of IgG^+^ plasma cells and TAMs and lower percentages of IgA^+^ plasma cells, fibroblasts and macrophages were observed in tumor tissues than in adjacent normal tissues in the SMC cohort (Fig. 1D). The differences in the distributions of T_REG_ cells, IgG^+^ plasma cells, TAMs and fibroblasts were validated in the KUL3 cohort. Regarding T cells, we did not observe similar distribution patterns of T_N_, T_H_, T_FH_, and T_CYTO_ cells, IELs and ILCs in the KUL3 cohort (Additional file 8: Fig. S1D).

### Revealing the cellular landscape of CRC in a large population using deconvolution analysis

**Fig. 2:**
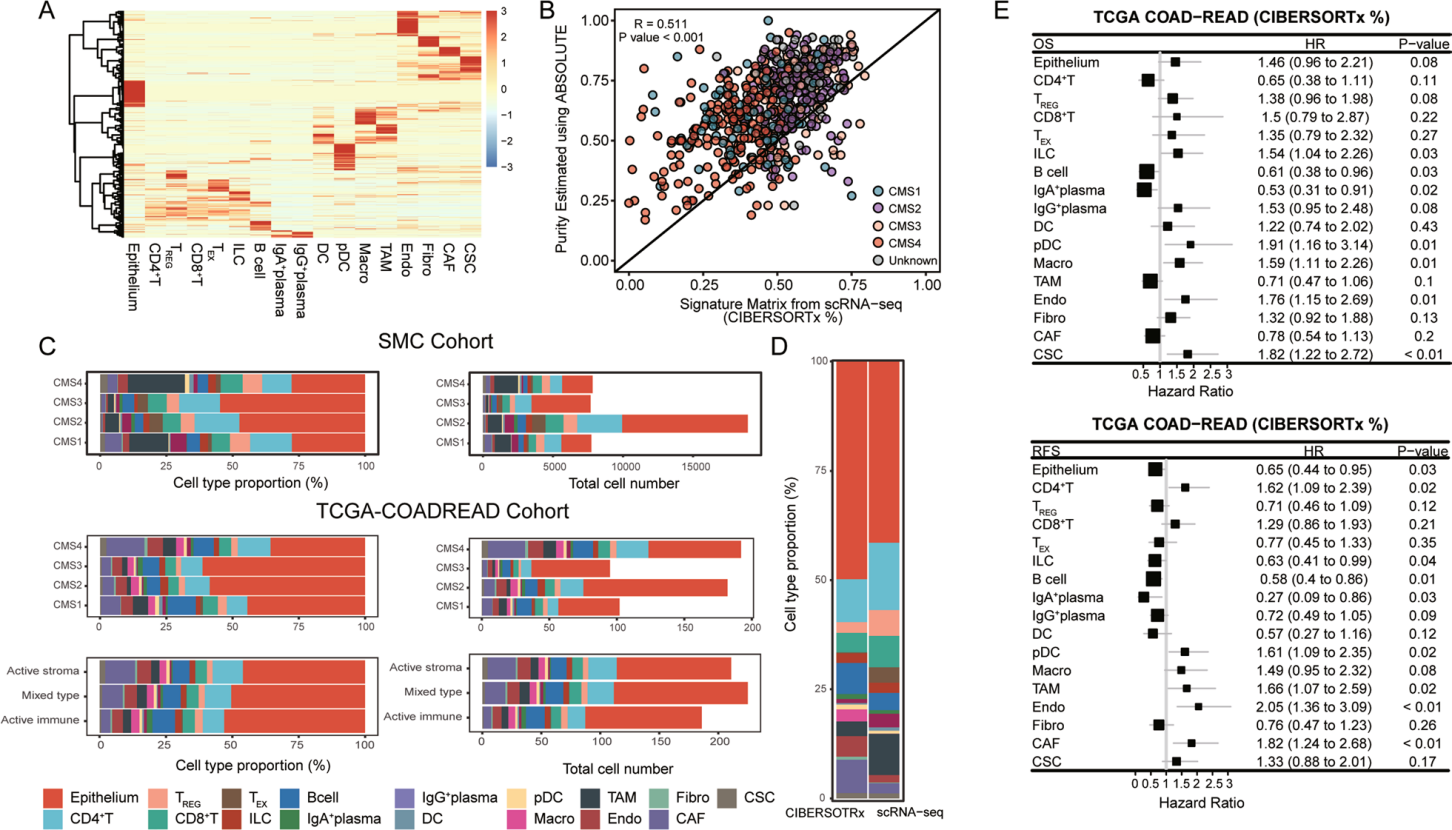
Cellular composition of CRC in a large population. **A**. Heatmap of the signature matrix used for CIBERSORTx. **B**. Correlation between the ABSOLUTE estimated purity and epithelial cell proportions inferred from CIBERSORTx for TCGA-COADREAD. The slope line, pearson correlation coefficient and p value are shown. Each point represents one sample colored by CMS. **C**. Proportions of identified cell clusters across CMSs in SMC (top), CMSs in TCGA-COADREAD (middle) and TME subtypes in TCGA-COADREAD (bottom) with the relative cell type proportions (left panel) and total cell numbers (right panel). **D**. Relative proportions of cell clusters inferred by CIBERSORTx in TCGA-COADREAD and from scRNA-seq data for SMC. The color legend is as in C. **E**. Forest plot of the hazard ratios (HRs) and p values for the association of cell infiltration levels with overall survival (top) and relapse-free survival (bottom). HR and p value were estimated using Cox proportional hazards regression model

To decipher the cellular composition of CRC in a larger population, we employed the newly developed deconvolution tool CIBERSORTx [27].Signature templates derived from the SMC cohort were created with simplified T-cell subgroups: CD4^+^ T cells, Treg cells, CD8 + T cells, T_EX_ cells and ILCs (Fig. 2A, Additional file 4: Table S4). The epithelial cell proportions of the SMC cohort skewed toward a low detection rate compared with tumor purity [22]. However, the epithelial cell proportions estimated with CIBERSORTx using our CRC-SRM exhibited a high correlation with the ABSOLUTE estimated purity in the cohort TCGA-COADREAD, indicating the reliability of our CRC-SRM (Fig. 2B). In addition, CIBERSORTx inferred cellular patterns among different consensus molecular subtypes (CMS: CMS1-4) [4] and our TME-driven subtypes (active immune (A.I.), active stroma (A.S.), and mixed types)), [5] revealing increased immune cells in the CMS1 and A.I. subtypes and higher stromal infiltration in the CMS4 and A.S. subtypes, consistent with previous reports (Fig. 2C bottom). However, these immune/stromal cell infiltration patterns of CMS1 and CMS4 were not observed in the SMC cohort (Fig. 2C top). T cells, B cells, TAMs, endothelial cells and CAFs constituted the TME ecosystem of CRCs (Fig. 2D). Increased abundances of epithelial cells, B cells, and stromal cells and fewer T cells and myeloid cells were observed using the cellular deconvolution method (Fig. 2D). Bias exists when inferring cellular compositions using scRNA-seq data. The discrepancy between the immune/stromal cell proportions was largely due to differences in cellular dissociation efficiencies, with stromal cells being more difficult to dissociate due to the extracellular matrix [42].

To assess the contributions of cellular lineages in shaping tumor biology, we assessed correlations of cellular proportions with patient survival data. TAMs, endothelial cells and CAFs were closely linked with unfavorable survival, whereas B cells and IgA^+^ plasma cells were associated with improved survival. T cells correlated weekly with survival (Fig. 2E). Overall, this analysis revealed the cellular landscape of CRCs and highlighted the pivotal role of myeloid lineages and stromal lineages in patient survival. To further understand the complex cellular heterogeneity of the EC and how epithelial cells orchestrate the MC to shape tumor phenotypes, we subsequently focused on deciphering tumor cell biodiversity and intercellular communication between the EC and MC.

### Assessment of transcriptional heterogeneity in colorectal tumor cells

**Fig. 3:**
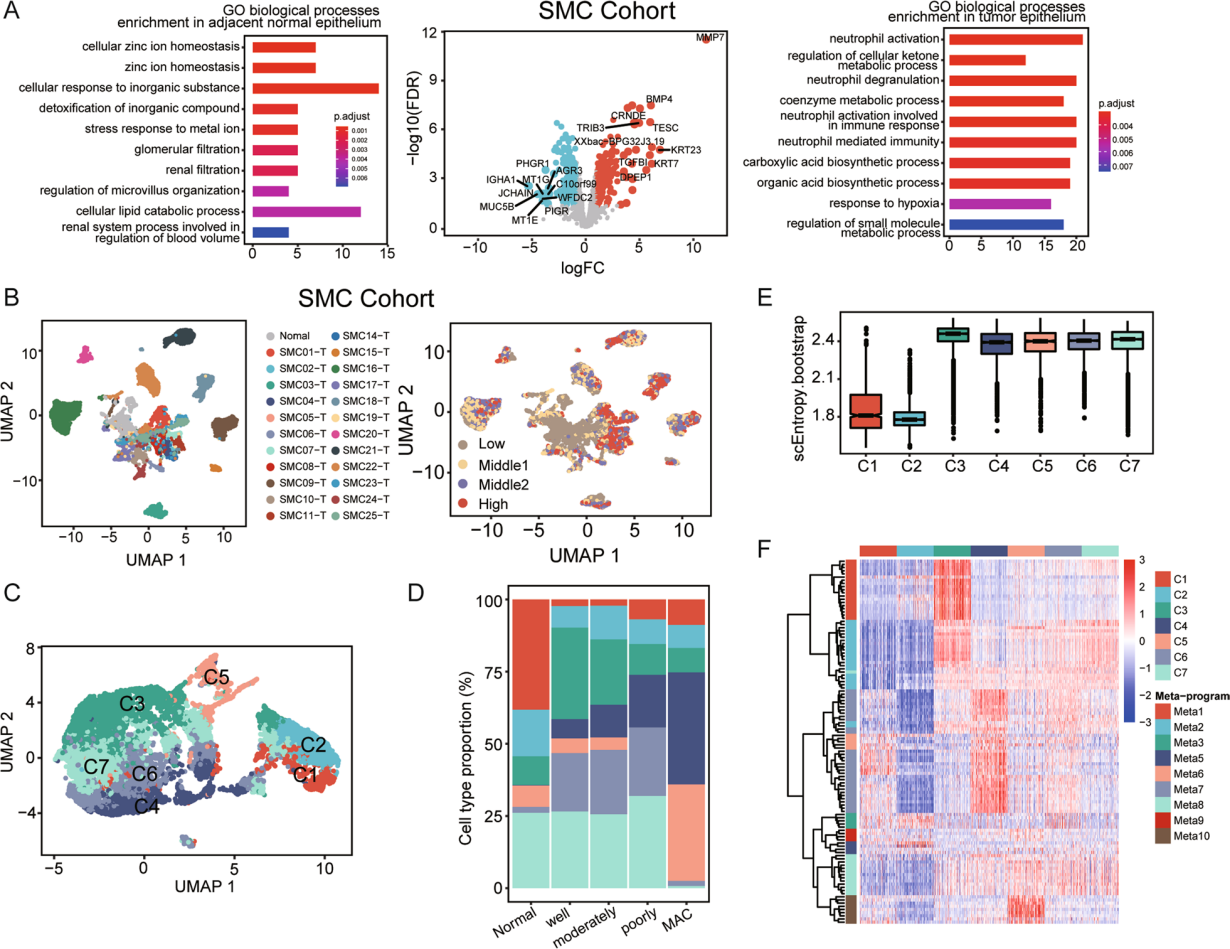
Functional heterogeneity among CRC epithelial cells in the SMC cohort. **A**. Volcano plot of differentially expressed genes between colorectal cancer cells and adjacent normal epithelial cells. Genes with FDR values less than 0.05 and absolute log2FC values greater than 1 are colored blue (upregulated in adjacent normal epithelial cells) or red (upregulated in cancer cells). GO enrichment plot is shown on each side, with the bar color indicating the enrichment significance, and bar length showing the number of common genes between the dysregulated genes and GO terms. **B**. UMAP plot of 18,561 epithelial cells from 23 patients colored by sample origin and CNV level. **C**. UMAP plot of epithelial cells using 115 cNMF-identified programs. Epithelial cell subclasses are annotated. **D**. Relative proportions of epithelial cell clusters in adjacent normal tissues and tumor samples of different pathological subtypes. Adenocarcinomas were graded as well, moderately and poorly differentiated. MAC: mucinous adenocarcinoma. **E**. Boxplot of scEntropy values across epithelial cell clusters. **F**. Hierarchical heatmap of the 115 expression programs in the SMC cohort. A total of 100 random cells in each cluster were chosen for visualization

Differential expression analysis between the tumor epithelium and adjacent normal epithelium identified 428 differentially expressed genes (DEGs) in the SMC cohort (Fig. 3A, Additional file 5: Table S5). The UMAP feature plot of the top 10 genes with the highest expression in the tumor epithelium confirmed these genes to be mainly expressed in the epithelium and not the microenvironment (Additional file 9: Fig. S2A). Gene Ontology (GO) terms regarding ion homeostasis and cellular lipid catabolic processes were enriched in normal epithelium; enrichment of the cellular response to hypoxia and neutrophil activation-related processes was detected in tumor epithelium. This observation was in line with our previous report [5].Similar alterations in cancer cells were observed in the KUL3 cohort (Additional file 10: Fig. S3A, Additional file 5: Table S5).

Consistent with previous reports, epithelial cells clustered according to patient origin, indicating the high intertumoral heterogeneity of CRC, which cannot be clearly explained by levels of copy number alteration (Fig. 3B, Additional file 10: Fig. S3B). To decipher the underlying transcriptomic features involved, cNMF analysis [29] was applied for each sample to identify its expression programs. In total, 123 expression programs were identified. Unsupervised clustering of these expression programs identified 10 metaprogrammes, including biological processes related to the cell cycle, translational initiation, inflammation, epidermal development, and neutrophil-related and protein biosynthetic processes (Additional file 9: Fig. S2B, Additional file 6: Table S6). Metaprogram 4 was excluded due to its irrelevance with other identified programs. Hierarchical clustering based on the remaining 115 expression programs divided the epithelial cells into 7 subtypes, namely, C1, C2, C3, C4, C5, C6 and C7 (Fig. 3C). C1-2 cells lacked reported cancer cell functional alterations (Additional file 9: Fig. S2C), suggesting that they are enriched in normal epithelial cells (Fig. 3D), and C1 cells expressed markers of mature colonocytes (*GUCA2B*, *SLC26A3* and *CA1*) (Additional file 9: Fig. S2D). All the evidence indicated that C1 and C2 cells are more similar to normal epithelial cells. In addition, the cell differentiation potential metric scEntropy [32] was much lower in C1-2 cells (Fig. 3E), indicating that C1-2 cells are terminally differentiated. C3 and C4 cells featured high proliferation and hypoxia markers, respectively (Fig. 3F, Additional file 9: Fig. S2B-C). Goblet cell markers such as *TFF3*, *SPDEF*, *SPINK4*, *REG4* and *AGR2* and biological functions related to O-glycan processing and glycoprotein biosynthetic processes were highly enriched in C5 cells, indicating that this cluster comprises goblet cell-like cells (Additional file 9: Fig. S2D). Consistently, C5 cells were mainly observed in mucinous adenocarcinoma tumor samples (Fig. 3D). C6 cells represented antigen-presenting cells owing to their high expression of MHC class II family genes (*HLA-DPA1*, *HLA-DPB1*, *HLA-DRA*, and *HLA-DRB1*) and *CD74* (Additional file 9: Fig. S2D). No specific gene signature was observed for C7 cells. C1-C7 cells were distributed across patients and tissue types, indicating that we identified common alternation states among patients. We validated the existing subclusters in the KUL3 cohort with similar tissue distributions (Additional file 10: Fig. S3C, D). Together, our identified epithelial clusters confirmed the heterogeneity of CRC tumor cells, and the clusters mainly varied in proliferation, hypoxia, antigen presentation, iron homeostasis maintenance and mucin production markers.

### C4 cells contribute to CRC progression

**Fig. 4:**
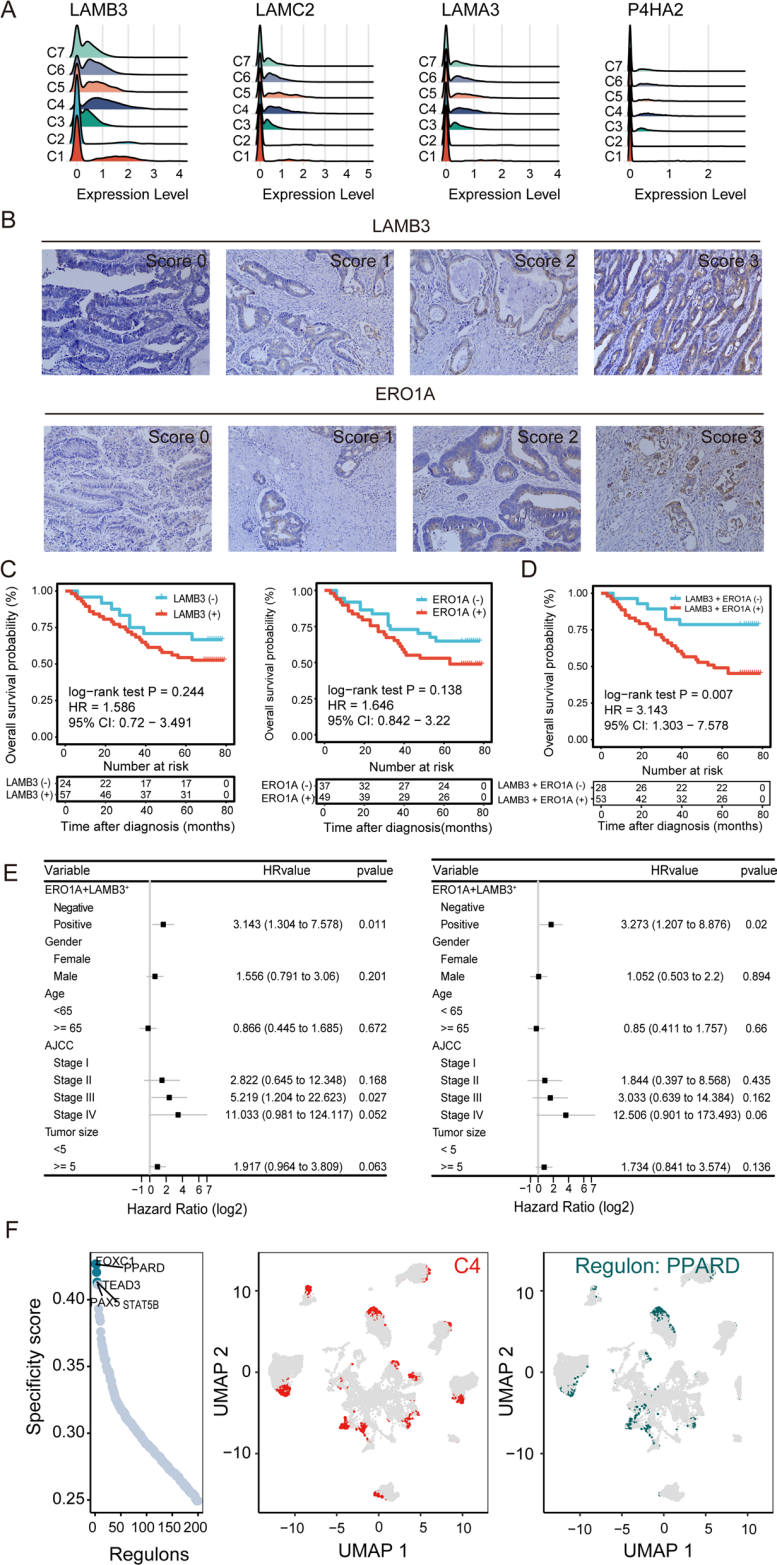
Validation of the invasive role of C4. **A**. Ridge plot of expression levels of partial EMT markers across epithelial subclasses. **B**. Representative IHC images of *LAMB3* (upper panel) and *ERO1A* (bottom panel) staining with the corresponding intensity score (0–3). All the images are at magnification 20 × . **C**. Overall survival curves for rectal cancer patients with LAMB3^+^ and LAMB3^−^ staining (left) and ERO1A^+^ and ERO1A^−^ staining (right) with sample size of each group placed on the bottom. HR, 95%CI and p value are annotated in text. **D**. Overall survival curves for rectal cancer patients with a LAMB3 + ERO1A^+^ or LAMB3 + ERO1A^−^ phenotype with sample size of each group placed on the bottom. HR, 95%CI and p value are annotated in text. **E**. Forest plot of univariate Cox (left) and multivariate Cox (right) analyses of the association of the LAMB3 + ERO1A^+^ or LAMB3 + ERO1A^−^ phenotype with overall survival. **F**. Ranks of regulons based on RSS (left) for the C4 group. The C4 group is highlighted in red (middle) and the activated regulon PPARD in dark green (right) in the UMAP plots

In general, invasion and metastasis of cancer cells are related to patient survival. To better understand the mechanism of CRC progression, we first explored the epithelial-mesenchymal transition (EMT) process. Canonical EMT markers, such as *ZEB1/2*, *TWIST1/2*, and *SNAIL1/SNAIL2,* were not detected in the EC (data not shown). [18] Some EMT markers, such as *LAMB3*, *LAMC2*, *LAMA3* and *P4HA2* [43] were detected in a few cells and mainly upregulated in C4 cells (Fig. 4A). Moreover, the relative abundance of C4 cells increased along with the cancer cell dedifferentiation level (Fig. 3D), indicating that C4 cells have high invasive potential. To validate the tumor-promoting role of C4 cells, we chose the EMT marker *LAMB3* and the C4 highly expressed gene *ERO1A* as surrogate markers. *LAMB3* has been reported to play a protumorigenic role in CRC through the AKT-FOXO3/4 axis [44].*ERO1A* has been reported to be an unfavorable factor in multiple cancers, including pancreatic cancer [45] and breast cancer, [46] with a cancer-promoting effect in HCT116 CRC cells by regulating integrin-β1. [47] However, the prognostic value of *ERO1A* in CRC has not been evaluated. The expression levels of *LAMB3* and *ERO1A* were assessed by IHC in tissue array 1, which includes 90 rectal cancer samples, and tissue array 3, which includes 30 colon cancer samples. Figure 4B shows the representative image of each staining score. The clinical characteristics are summarized in Table 1. Higher expression of *LAMB3* or *ERO1A* alone was not associated with patient prognosis (Fig. 4C). However, when *LAMB3* and *ERO1A* were considered together as surrogate markers, their increased expression was significantly related to adverse prognosis (Fig. 4D). In addition, C4 cells (*LAMB3*^+^*ERO1A*^+^) were found to be a risk factor independent of TNM stage (Fig. 4E). Thus, C4 cells exhibited hypoxia and partial EMT markers and were closely related to poor differentiation, invasion and short survival.

To assess the underlying regulatory networks contributing to the invasive phenotypes of C4 cells, we employed SCENIC to identify the transcription factors (TFs) responsible [30].The top 5 TFs with the highest regulon specificity score (RSS), which was calculated based on an entropy-based strategy [28], were considered master regulators of the clusters. We found *PPARD*, which had the highest RSS and has been reported to accelerate CRC progression, [48] to be activated in C4 cells (Fig. 4F), suggesting that it might drive the invasive properties of cancer cells.

### The parenchymal cell pattern is closely linked with environmental cell composition

**Fig. 5:**
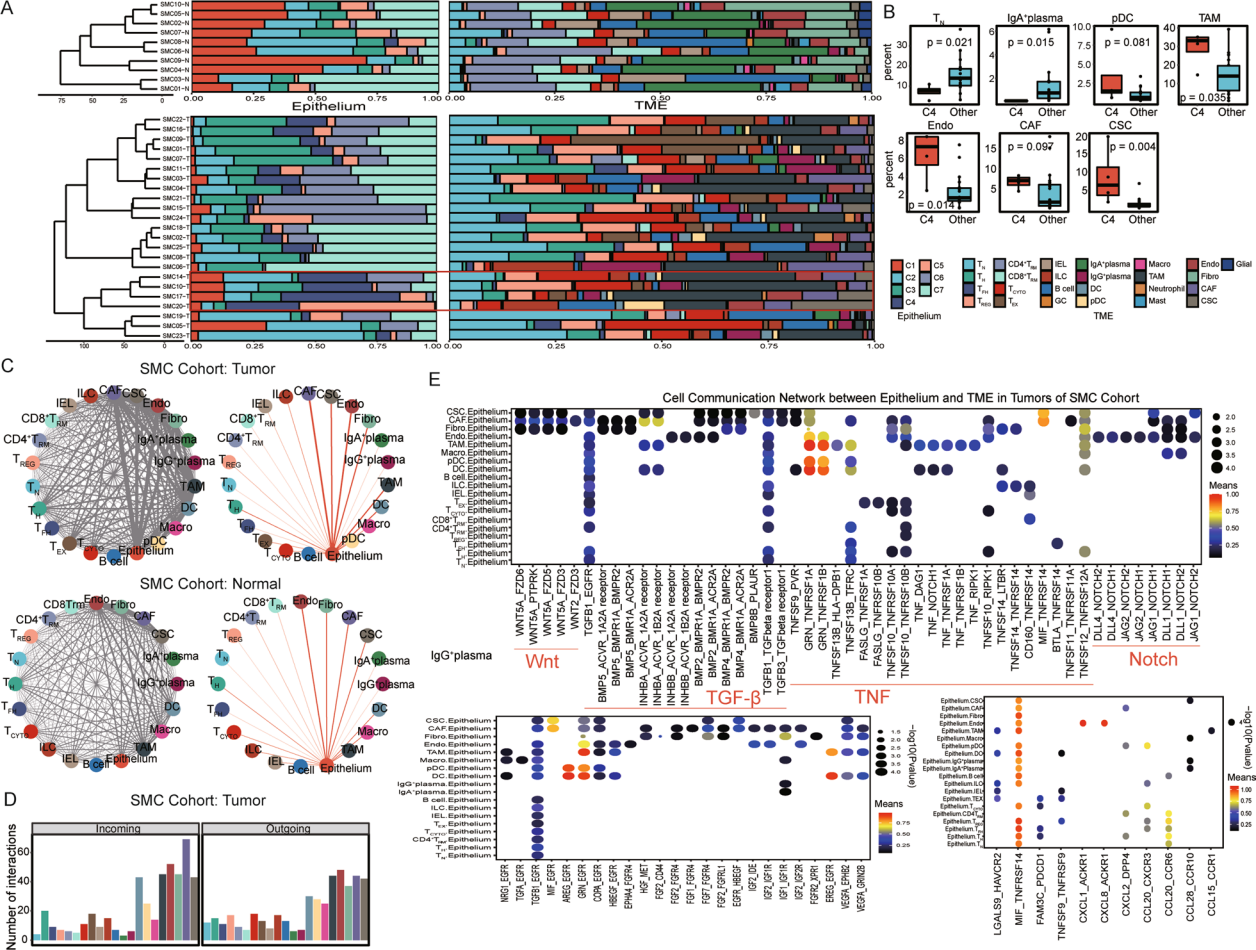
Dynamic interactions between the EC and MC in the SMC cohort. **A**. Relative proportions of epithelial cell clusters and TME cell clusters. Samples were clustered according to the distribution pattern of epithelial cells with the corresponding TME cell cluster distribution in the right panel. **B**. Frequencies of selected cell types for Group C4 and the other groups. The Wilcoxon rank-sum test p value is shown. **C**. Cell‒cell interactions in tumors (upper panel) and adjacent normal tissues (bottom panel) identified by CellPhoneDB (left). Interactions between cancer cells and nonmalignant cells are highlighted (right). A thicker edge represents more interactions. **D**. Bar plot of the incoming and outcoming events for epithelial-microenvironment communications in tumors. **E**. Bubble plots of ligand‒receptor pairs between cancer cells and nonmalignant cells in tumors. The corresponding category is annotated. Dot size and color represent the enrichment score and relative mean expression level of ligand‒receptor pairs, respectively

To unveil interactions between the epithelium and its surrounding microenvironment, we first explored cellular composition similarities between the EC and MC. Unsupervised hierarchical clustering of epithelial cells revealed one group, Group C4, containing a considerable C4 cell proportion (Fig. 5A, highlighted in the red rectangle). The epithelial cell cluster composition patterns of the SMC19-T, SMC05-T and SMC23-T samples were similar to those of adjacent normal tissues (Fig. 5A, upper panel), indicating the biological properties of these SMC samples to be less malignant. Similar differences in cellular compositions were observed between the tumor core and tumor border, indicating less intratumor heterogeneity than intertumoral heterogeneity in CRC (Additional file 11: Fig. S4A). The cellular composition of the surrounding microenvironment was tremendously different between Group C4 and the other groups. TAM, pDC and stromal lineages (endothelial cells, CAFs and CSCs), which were associated with poor survival (Fig. 2E), were more abundant in Group C4 (Fig. 5B). Thus, aggressive cancer cells can orchestrate unfavorable and immunosuppressive microenvironments to promote cancer progression.

To better understand the relationship between the epithelium and microenvironment, we employed CellPhoneDB [33, 34] to construct a cell‒cell communication network. More extensive communication was identified in tumors than in adjacent normal tissues (Fig. 5C), demonstrating that cells in tumors interact closely to promote tumorigenesis and progression. The cell‒cell interaction network was composed of epithelial cells, myeloid cells (DCs, pDCs, macrophages and TAMs) and stromal cells (myofibroblasts, CAFs, fibroblasts and endothelial cells) (Fig. 5C), and the EC-MC network was dominated by myeloid cells and stromal cells in both tumor and adjacent normal tissues (Fig. 5C, D, Additional file 11: Fig. S4B-E). The dominant role of myeloid and stromal cells was consistent with what is seen in other cancers. [49] To systematically decipher the crosstalk between the epithelium and microenvironment, specific ligand‒receptor pairs were further investigated. Potential interactions involving canonical oncogenic signaling pathways, including the b-catenin/Wnt, transforming growth factor-beta (TGFB), tumor necrosis factor (TNF) and Notch signaling pathways, were assessed (Fig. 5E, Additional file 11: Fig. S4F). Stromal cells were found to secrete various members of the Wnt gene family (*WNT4*, *WNT5A*, and *WTN2*) and the TGF-beta superfamily; myeloid cells were found to secrete TNF ligand family members (*TNFSF9*, *TNFSF10*, *TNFSF11*, and *TNFSF12*). The Wnt, TGF-beta and TNF signaling pathways are critical for maintaining cell stemness, promoting cell invasion and causing cell inflammation. Their corresponding receptors were detected on the tumor epithelium. In addition, various growth factors were secreted by the two cell lineages to support tumor growth. Conversely, no specific functional patterns were found in terms of immune checkpoints, costimulatory molecules and chemokines.

### Diverse TAM subtypes are present in CRC

**Fig. 6:**
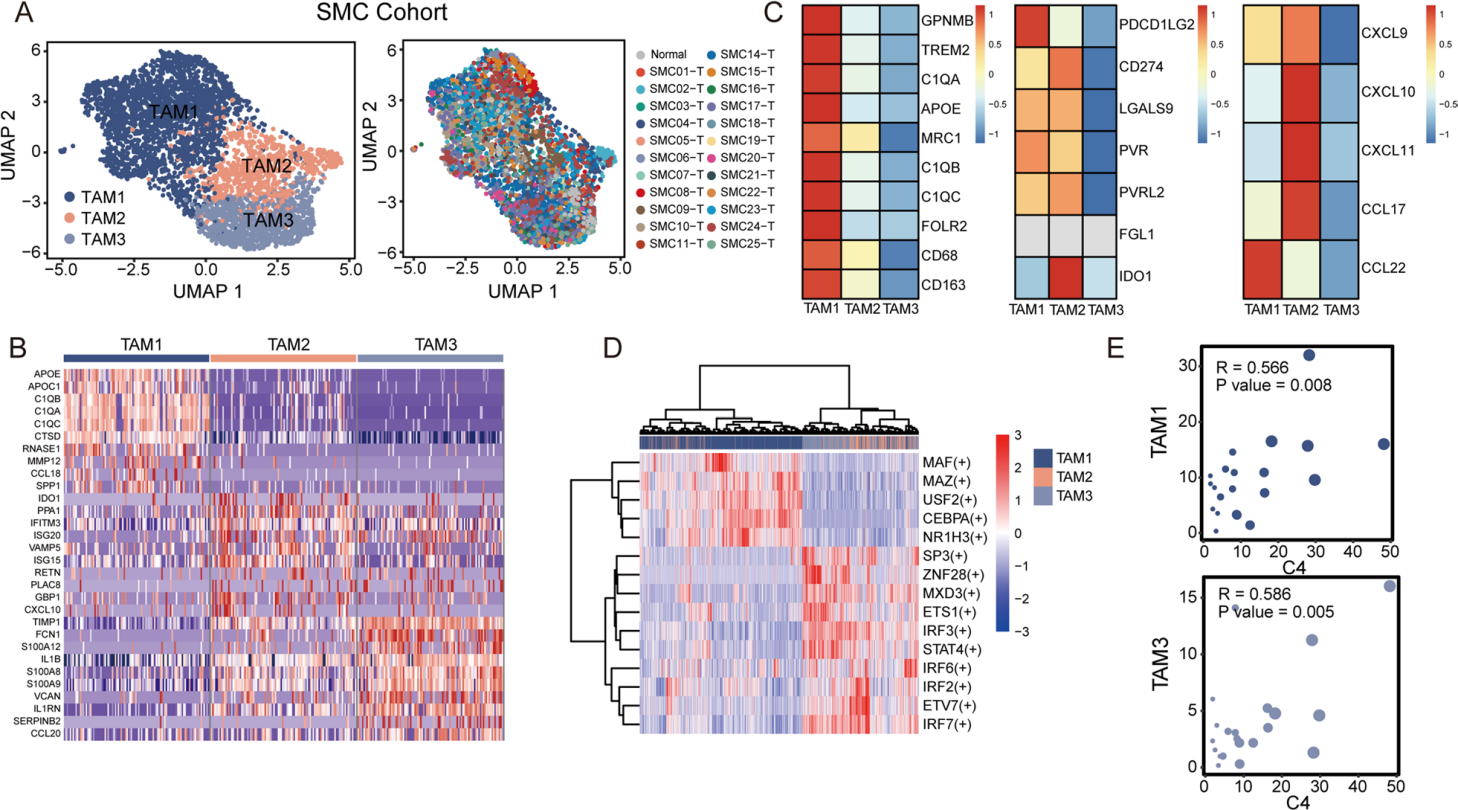
Characterization of myeloid cells by scRNA-seq in the SMC cohort. **A**. UMAP plot of TAMs colored by cell cluster and patient origin. **B**. Heatmap of marker genes identified through Seurat. For each cell cluster, cells were downsampled to 100. **C**. Heatmap of well-known macrophage-related immunosuppressive, immune checkpoint and chemokine gene expression across TAMs. **D**. Heatmap of the top 5 ranked regulons in TAM1-3. **E**. Dot plot of the correlation between the proportions of C4 cells in epithelial cells and TAM1 (top) or TAM3 (bottom) in the TME. Each dot represents a patient, and a larger size indicates a higher C4 cell proportion. Correlation test is determined using Pearson test

We then further analyzed myeloid and stromal lineages. According to macrophage classification in lung cancer, [50] 5,246 TAMs in the SMC cohort were categorized into three subtypes: TAM1, TAM2, and TAM3 (Fig. 6A). For the KUL3 cohort, we also identified one cluster, herein termed TAM4, that showed high enrichment of the MMP family (MMP1, MMP9 and MMP12) (Additional file 12: Fig. S5A). Significant marker genes were identified (Fig. 6B, Additional file 12: Fig. S5B, Additional file 3: Table S3). Notably, the TAM1 subtype was characterized by high expression of genes related to an immunosuppressive phenotype (*APOE*, *C1QC*, *GPNMB*, *CD163* and *TREM2*) (Fig. 6C). The TAM2 and TAM3 subtypes showed enrichment of proinflammatory signaling molecules (*FCN1*, *S100A8*, *S100A9*, *VCAN* and *IL1B*) (Fig. 6B, Additional file 12: Fig. S5B). The TAM2 subtype was considered separately due to its high expression of chemokine-related genes (*CXCL10* and *CXCL11*), which have been reported to promote T-cell infiltration [51].Interferon-stimulated genes (*ISG15* and *ISG20*) and the guanylate-binding family protein *GBP1*, which are induced in IFN-g-activated macrophages, were enriched (Fig. 6B, Additional file 12: Fig. S5B), indicating their potential antitumor activity. However, the TAM2 subtype also inhibits T-cell activation by upregulating immune checkpoint inhibitors (*CD274* and *PVR*) and *IDO1*, which have been widely reported to exert immunosuppressive effects by activating Treg cells [52].Thus, the TAM2 subtype appears to bridge the innate and adaptive immune responses and is functionally heterogeneous (Fig. 6C). The TF gene expression pattern identified by SCENIC clearly clustered TAMs into two subgroups: TAM1 with TAM4 (Additional file 12: Fig. S5C) and TAM2 with TAM3 (Fig. 6D, Additional file 12: Fig. S5C). This result indicates that TAMs in CRC have two branches, immunosuppressive and proinflammatory. Moreover, cell type frequencies of the TAM1 and TAM3 subtypes correlated positively with the C4 cell proportion in the epithelium in the SMC cohort (Fig. 6E). In contrast, macrophages, which mainly infiltrated adjacent normal tissues, correlated inversely with the C4 cell proportion in the KUL3 cohort (Additional file 12: Fig. S5D). The relationship between C4 cells and the TAM1 subtype was consistent in the SMC and KUL3 cohorts. However, we did not observe significant correlations between the TAM3 subtype and C4 cells in the KUL3 cohort. These data support that TAMs might foster tumor cell invasion by producing an immunosuppressive environment.

### Diverse stromal subtypes are present in CRC

We next focused on stromal lineages, with no further subclassification performed for endothelial cells. Fibroblasts, CAFs and CSCs were subclassified into 10 classes, with only one class (S10) discovered in the KUL3 cohort (Additional file 13: Fig. S6A, Additional file 14: Fig. S7A). Subclasses were annotated according to the classification system provided by Lambrechts et al. [19] S1-4 and S10 were all exclusive to adjacent normal tissue (Additional file 13: Fig. S6A, Additional file 14: Fig. S7A). The marker genes in each cluster are shown in Additional file 13: Fig. S6B and Additional file 14: Fig. S7B (Additional file 3: Table S3). Specifically, S1 and S2 featured adipocyte markers (*CFD* and *APOD*), resembling the phenotype of lipofibroblasts in lung tissue [53]. S3 contained mesenchymal cells located in the colon lamina propria (*APOE* and *ADAMDEC1* markers), and S10 was characterized by coexpression of *KCNN3* and *P2RY1*, which have been reported to regulate multiple neuromuscular transmission processes in the colon [19].S4 expressed Wnt signaling genes (*FRZB*), *SOX6* and *PDGFRA*, which function in maintaining the epithelial stem cell niche. Previous studies have described *PDGFRA*^+^ fibroblasts as progenitors that give rise to lipofibroblasts and myofibroblasts [54].The differentiation trajectory of fibroblast lineages in our study also indicated that S4 include highly plastic cells with the potential to give rise to other subtypes (Additional file 13: Fig. S6C, Additional file 14: Fig. S7C). For CAFs, a minor subclass (S6) characterized by *SERPINE1, IGF1*, *WT1* and *KRT19* expression was isolated. *IGF1* has been reported to be associated with survival in bladder urothelial carcinoma, [41] and higher expression of *SERPINE1* has also been reported to be an adverse factor in lung cancer[50] .In addition, S6 expressed *KRT19* and *WT1*, indicating that S6 resembles the mesothelial phenotype [55].The remaining CAF subgroup (S5) was characterized by enrichment of collagens (*COL12A1*, *COL1A1* and *COL3A1*), *INHBA* and MMPs (*MMP1* and *MMP11*) (Additional file 13: Fig. S6B, Additional file 14: Fig. S7B). SCENIC analysis also identified unique TFs for S6, indicating that the underlying molecular network of S5 is completely different from that of S6 (Additional file 13: Fig. S6D, Additional file 14: Fig. S7D).

CAFs have been reported to generate a modified extracellular matrix (ECM) environment to promote cancer cell survival. Consistently, patients with high infiltration levels of S5 and S6 subtype cells showed worse survival (Additional file 13: Fig. S6E). The correlation of the C4 cell proportion with the frequency of CAFs indicated that the S5 subtype contributes to an aggressive phenotype of the epithelium (Additional file 13: Fig. S6F, Additional file 14: Fig. S7E). With regard to the contractile genes (*ACTA2* and *TAGLN*) expressed by CSCs, we identified three classes (S7-9). S7 corresponded to pericytes because of its high expression of the characteristic genes *RGS5*, collagens (*COL4A1*, *COL4A2* and *COL18A1*) and *NOTCH3* (which is related to vessel maturation). S8 was deemed myofibroblasts and was characterized by smooth muscle-related contractile genes (*MYH11* and *PLN*), while S9, which represented smooth muscle cells, coexpressed contractile genes and cytoskeletal genes (*MYH11*, *SYNPO2*, *CNN1* and *DES*) (Additional file 13: Fig. S6B. Fig. S7B). Overall, our analysis of colorectal stromal cells suggests that S5 subtype cells play a role in promoting cancer progression.

### The infiltration level of TAM1 is related to C4 and is associated with worse survival

**Fig. 7:**
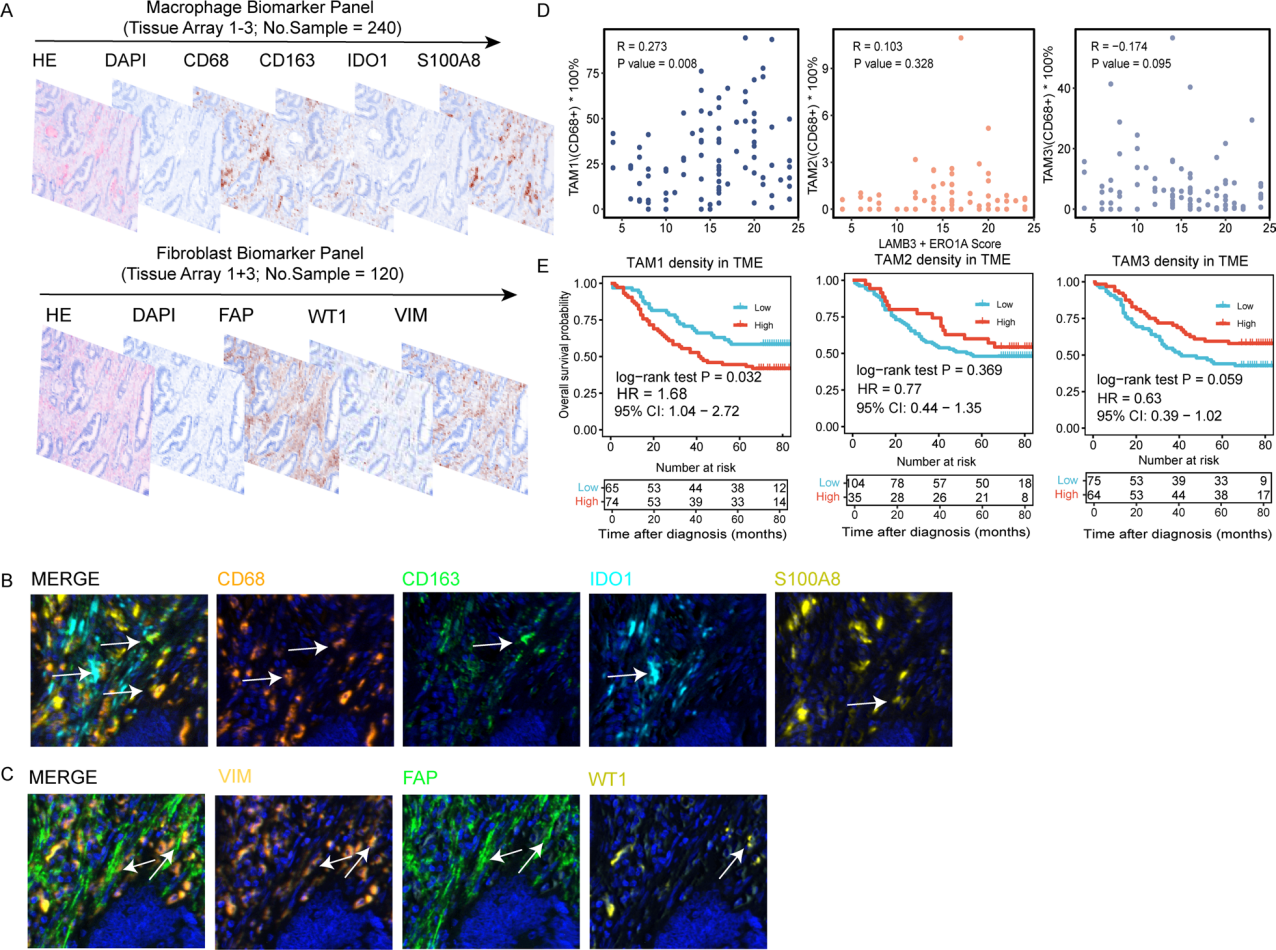
Validation of the orchestrated roles of TAM1 and C4 cells. **A**. Multiplex IHC designed for the macrophage biomarker panel and fibroblast panel. **B**. Representative multicolor IHC staining for *CD68*^+^*CD163*^+^ *IDO1*^−^ *S100A8*^−^ TAM1, *CD68*^+^*CD163*^−^ *IDO1*^+^ *S100A8*^−^TAM2 and *CD68*^+^*CD163*^−^ *IDO1*^−^ *S100A8*^+^ TAM3. **C**. Representative multicolor IHC staining of *VIM*^+^ *FAP*^+^ *WT1*^−^S5 and *VIM*^+^*FAP*^+^*WT1*^+^S6 cells. **D**. Dot plot of the correlation between the infiltration level of TAM1-3 with the surrogate score of C4 cells in tissue arrays 1 and 3. Each dot represents a CRC patient sample. Correlation test is determined using Pearson test. **E**. Overall survival curves for TAM1-3 in tissue arrays 1 and 2 with sample size of each group placed on the bottom. HR, 95%CI and p value are annotated in text

Considering the important role of TAMs and CAFs in the progression of CRC, we next evaluated the relative abundances of TAM1-3 and S5-6 cells to investigate their clinical value in our IHC validation cohort. Detailed clinical characteristics are summarized in Table 1. M panel and F panel were designed for multiplex IHC according to the genes highly expressed in TAMs and CAFs (Fig. 7A). *CD68*^+^*CD163*^+^*IDO1*^−^*S100A8*^−^, *CD68*^+^*CD163*^−^*IDO1*^+^*S100A8*^−^and *CD68*^+^ *CD163*^−^*IDO1*^−^*S100A8*^+^ TAMs were considered TAM1, TAM2 and TAM3, respectively (Fig. 7B). For CAFs, *VIM*^+^*FAP*^+^ *WT1*^−^ and *VIM*^+^*FAP*^+^*WT1*^+^ were deemed S5 and S6 cells, respectively (Fig. 7C). Consistent with the analysis of the SMC and KUL3 cohorts, the infiltration level of TAM1 correlated positively with the C4 surrogate marker. No relationship was observed between TAM2 or TAM3 and C4 cells (Fig. 7D). Moreover, the TAM1 infiltration level in the TME correlated with adverse overall survival (p = 0.032). Patients with high TAM2 or TAM3 infiltration tended to have a favorable prognosis, though the correlations were not significant (Fig. 7E). We did not observe any prognostic value for S5 and S6 cells.

## Discussion

Currently, the immune landscape and TME heterogeneity in CRC had been well characterized in single-cell transcriptome studies [11, 21, 22]. Indeed, widespread cellular heterogeneity not only exists in the composition of the CRC microenvironment but can also be observed in the cancer cell compartment. Tumor cells in glioblastoma exhibited diverse functional activity in oncogenic signaling, proliferation, complement/immune response, and hypoxia [56]. And stemness program expression level had been reported to be related to the heterogeneity of ovarian cancer cells [57]. So far, 14 cancer-related functional states had been summarized from 41, 900 cells in 25 human cancers [58]. It is worth knowing that tumor cells’ intrinsic biological features determine cancer growth, invasion and metastasis. Shanzhao Jin et al. discovered one malignant cluster with epithelial–immune dual feature that was related to poor survival [49]. In this study, we integrated single-cell transcriptomes, bulk RNA-seq data, and IHC in vitro validation to comprehensively decode the cancer cellular heterogeneity and its crosstalk with TME, hoping to gain deep insight into the CRC’s ecosystem, a flow chart shows the design of our study (Additional file 15: Fig. S8).

Cell proliferation, hypoxia and inflammatory reactions related pathways were highly activated in CRC cancer cells. Notably, we discovered one cancer cell subgroup, C4, exhibited hypoxia and partial EMT markers, closely associated with TAM, CAF and adverse prognosis. The roles of TAMs and CAFs in inducing immunotherapy resistance, [59, 60] promoting chemotherapy resistance [61] and supporting tumor growth [41] have been elucidated in various cancer types. It had been reported that CD163 + Tim4 + macrophages resided in omentum form a protective niche to promote ovarian tumor spread [62]. In addition, myeloid cells had been reported to initiate tumor formation by releasing reactive oxygen species to drive genomic damage. [63] The outstanding pro-oncogenic effect of TAM attract much attention recently, and immunotherapy targeting myeloid cells had been conducted in CRC [11].Our finding underscored that TAM contributes to the aggressive phenotype of cancer cells, which is C4 in CRC, by collaborating with CAF. It is worth noting that cancer cells and CAF had been reported to take charge of releasing chemokine to recruit monocyte into TME [64, 65]. Our findings support a central role of C4 cells, TAMs and CAFs in the whole EC-TME communication networks. Therefore, we speculated that drugs simultaneously targeting both cancer and microenvironment cells or disrupting this central communication network are very promising in the further. C4 cells, TAMs or more precisely, TAM1 and CAFs warrant further investigation.

This study depicts cancer cell heterogeneity at the single-cell level and comprehensively describes the connection between epithelial composition and microenvironment cell infiltration patterns in CRC. In summary, our work helps to deepen our understanding of the CRC ecosystem, elaborates on the complicated cooperation between cancer cells and the TME and provides a solid foundation for developing drugs targeting C4 cells, TAMs and CAFs. Nevertheless, there are several limitations in our study that we must acknowledge. First, a previous study emphasized the role of neoantigens, in cooperation with immune cells, in driving lung cancer evolution [66].Due to the technical limitations of scRNA-seq, genomic alteration data were not included in our analysis to decipher the heterogeneity of epithelial cells. Second, the cell‒cell communication networks between microenvironment cells and the interactions of microenvironment cells that form an immunosuppressive environment are of importance to CRC oncogenesis and progression. As this was not the focus of our study, we did not explore their role in shaping the CRC landscape. Third, although the epithelium-microenvironment communication network inferred by CellPhoneDB provided solid evidence for a dominant role of TAMs and CAFs, the infiltration levels of which were closely linked with an aggressive phenotype of epithelial cells, expression levels of the majority of identified ligands and receptors were not distinct between C3-C7 cells (data not shown). Fourth, the tumor-promoting role of C4 cells and TAM1 was only validated by evaluating expression levels, and further functional biological validation in experiments such as transwell-invasion assay to evaluate C4’s invasiveness and coculture of C4 cells and TAM1 are necessary to evaluate tumor reactivity.

## Supplementary Information

**Additional file 1: Table S1.** Basic clinical information and QC metric of SMC and KUL3 cohort.

**Additional file 2: Table S2.** Cellular annotation and clinical characteristic of SMC and KUL3 cohort. Relate to Figure 1 and Figure S1.

**Additional file 3: Table S3.** Representative marker genes used for cell type annotation.

**Additional file 4: Table S4.** Signature matrix for CIBERSORTx and infered cellular fractions of TCGA-COADREAD. Related to Figure 2.

**Additional file 5: Table S5.** Biological functional difference between colorectal cancer cells and adjacent normal epithelia in SMC and KUL3 cohort. Related to Figure 3A and S3A.

**Additional file 6: Table S6.** Identified meta-programs and its biological process annotation. Related to Figure 3F, Figure S2B.

**Additional file 7: Table S7.** Key resource table.

**Additional file 8: Figure S1.** Cellular landscape of CRC in the KUL3 cohort. **A**. UMAP plot of 26,268 cells colored by cell cluster, CMS and sample origin. Each dot represents a cell and cellular cluster is annotated with text. **B**. Proportions of the identified cell clusters distributed across tumor, border and adjacent normal tissues with the relative cell type proportions and total cell numbers. Upper: all cell clusters; lower: immune and stromal cell clusters. **C**. Heatmap of representative markers for the cell clusters. A total of 100 random cells in each cluster were chosen for visualization. The color legend is as in B. **D**. Frequencies of the selected cell types for tumor, border and adjacent normal samples. The Kruskal-Wallis test p value is shown.

**Additional file 9: Figure S2.** Functional heterogeneity of CRC epithelial cells in the SMC cohort. **A**. UMAP feature plot of the top 10 upregulated genes in the SMC cohort. **B**. Correlation heat map of 123 programs identified by cNMF. The Pearson correlation coefficient is indicated by the color. **C**. Heatmap of the relative mean signature score of cancer cell functional signatures across C1-C7. The signature score was calculated using the “AUCell” package. **D**. Heatmap of functional genes across C1-C7. A total of 100 random cells in each cluster were used for visualization

**Additional file 10: Figure S3.** Epithelial heterogeneity validation in KUL3 cohort. **A**. Volcano plot of the differentially expressed genes between colorectal cancer cells at the core (up) or border (down) and adjacent normal epithelial cells. Genes with FDR values less than 0.05 and absolute log2FC values greater than 1 are colored blue (upregulated in adjacent normal epithelial cells) or red (upregulated in cancer and border tissues). GO enrichment plot is shown on each side with the bar color indicating enrichment significance, and bar length showing the number of overlapping dysregulated genes and the GO term. **B**. UMAP plot of 6,178 epithelial cells from 6 patients colored by patient and tissue type (tumor, border and normal). **C**. Hierarchical heat map of 115 expression programs. The cells in each cluster were down-sampled to 100. The corresponding meta-programs are listed in the rows. **D**. Relative proportions of epithelial cell clusters for adjacent normal tissues and colorectal adenocarcinoma samples of different histological grades

**Additional file 11: Figure S4.** Dynamic interactions between the EC and MC. **A**. Relative proportions of epithelial cell clusters and TME cell clusters in the KUL3 cohort. Samples were clustered according to the distribution pattern of epithelial cells with the corresponding TME cell cluster distribution shown in the right panel. **B**–**E.** Bar plot showing the number of incoming events and outgoing events for epithelial cells communicating with TME cells in normal tissues of the SMC cohort (**B**), tumor tissues of the KUL3 cohort (**C**), border tissues of the KUL3 cohort (**D**) and normal tissues of the KUL3 cohort (**E**). **F**. Bubble plots of ligand-receptor pairs between epithelial cells and TME cells in normal tissues of the SMC cohort. Dot size and color represent the enrichment scores and the relative mean expression level of ligand-receptor pairs, respectively.

**Additional file 12: Figure S5.** Characterization of myeloid cells in the KUL3 cohort. **A**. UMAP plot of myeloid cells colored by cell cluster and sample origin. **B**. Heatmap of marker genes identified through Seurat. For each cell cluster, cells were down-sampled to 100.** C**. Heatmap plot of the top 5 ranked regulons in TAM1-TAM4 identified by pySCENIC. **D**. Dot plot of the correlation between the proportions of C4 cells in epithelial cells and macrophages (left), TAM1 (middle) and TAM3 (right) in the TME. Each dot represents a patient, and a larger dot size means a higher C4 cell proportion.

**Additional file 13: Figure S6.** Characterization of stromal cells in the SMC cohort. **A**. UMAP plot of stromal cells colored by cell cluster and sample origin. **B**. Heat map of marker genes identified through Seurat. For each cell cluster, cells were down-sampled to 100. **C**. Differentiation trajectories inferred by Monocle2. Dots represent stromal cells and are colored by identified cell cluster. **D**. Heat map of the top 5 ranked regulons in S5 and S6. **E**. Relapse-free survival curves for S5 (top) and S6 (bottom) in the TCGA-COADREAD cohort. **F**. Dot plot of the correlation between the proportions of C4 cells in epithelial cells and S5 in the TME. Each dot represents a patient, and a larger size means a higher C4 cell proportion. Correlation test is estimated by Pearson correlation test.

**Additional file 14: Figure S7.** Characterization of stromal cells in the KUL3 cohort. **A**. UMAP plot of stromal cells colored by cell cluster and sample origin. **B**. Heat map of marker genes identified through Seurat. For each cell cluster, cells were down-sampled to 100. **C**. Differentiation trajectory inferred by Monocle2. Dots represent stromal cells colored by identified cell cluster. **D**. Heat map plot of the top ranked regulons. **E**. Dot plot of the correlation between the proportions of C4 cells in epithelial cells and S5 cells in the TME. Each dot represents a patient, and a larger size means a higher C4 cell proportion. Correlation test is estimated by Pearson correlation test.

**Additional file 15: Figure S8.** Workflow of this study. Single-cell transcriptomes and bulk RNA-seq data were integrated to fully analyze the complicated relationship between colorectal epithelium and surrounding environment. C4 cells were featured with high invasive potential and related with TAMs and CAFs, and further validated in vitro using IHC and mIHC.

## Data Availability

The cohorts SMC and KUL3 are accessible through the GEO database (https://www.ncbi.nlm.nih.gov/geo) under accession numbers GSE132465 and GSE144735. The cohort TCGA-COADREAD was downloaded through the UCSC Xena browser (https://xenabrowser.net). IHC antibodies and software used in this work are presented in Additional file 7: Table S7.

## Abbreviations

CAF: Cancer-associated fibroblast
cNMF: Consensus nonnegative matrix factorization
CPM: Counts per million
CRC: Colorectal cancer
DC: Dendritic cell
DEG: Differentially expressed gene
EC: Epithelium compartment
FPKM: Fragments per kilobase million
GC: Germinal center
GO: Gene ontology
IEL: Intraepithelial lymphocyte
ILC: Innate lymphoid cell
MC: Microenvironment compartment
NK: Natural killer cell
PCA: Principal component analysis
RSS: Regulon specificity score
sc-RNAseq: Single-cell RNA sequencing
SNN: Shared nearest neighbor
TAM: Tumor-associated macrophage
T_CYTO_: Cytotoxic T cell
T_EX_: Exhausted T cell
TF: Transcription factor
T_FH_: T follicular helper cell
T_H_: T helper cell
TME: Tumor microenvironment
T_N_: Naïve T cell
TPM: Transcripts per million
T_REG_: Regulatory T cell
T_RM_: Tissue-resident T cell
UMAP: Uniform manifold approximation and projection
