# Supplementary figures and images for: Decoding the colorectal cancer ecosystem emphasizes the cooperative role of cancer cells, TAMs and CAFsin tumor progression
Source: J Transl Med. 2022 Oct 8;20:462. doi: 10.1186/s12967-022-03661-8 (PMC9548187; doi:10.1186/s12967-022-03661-8)

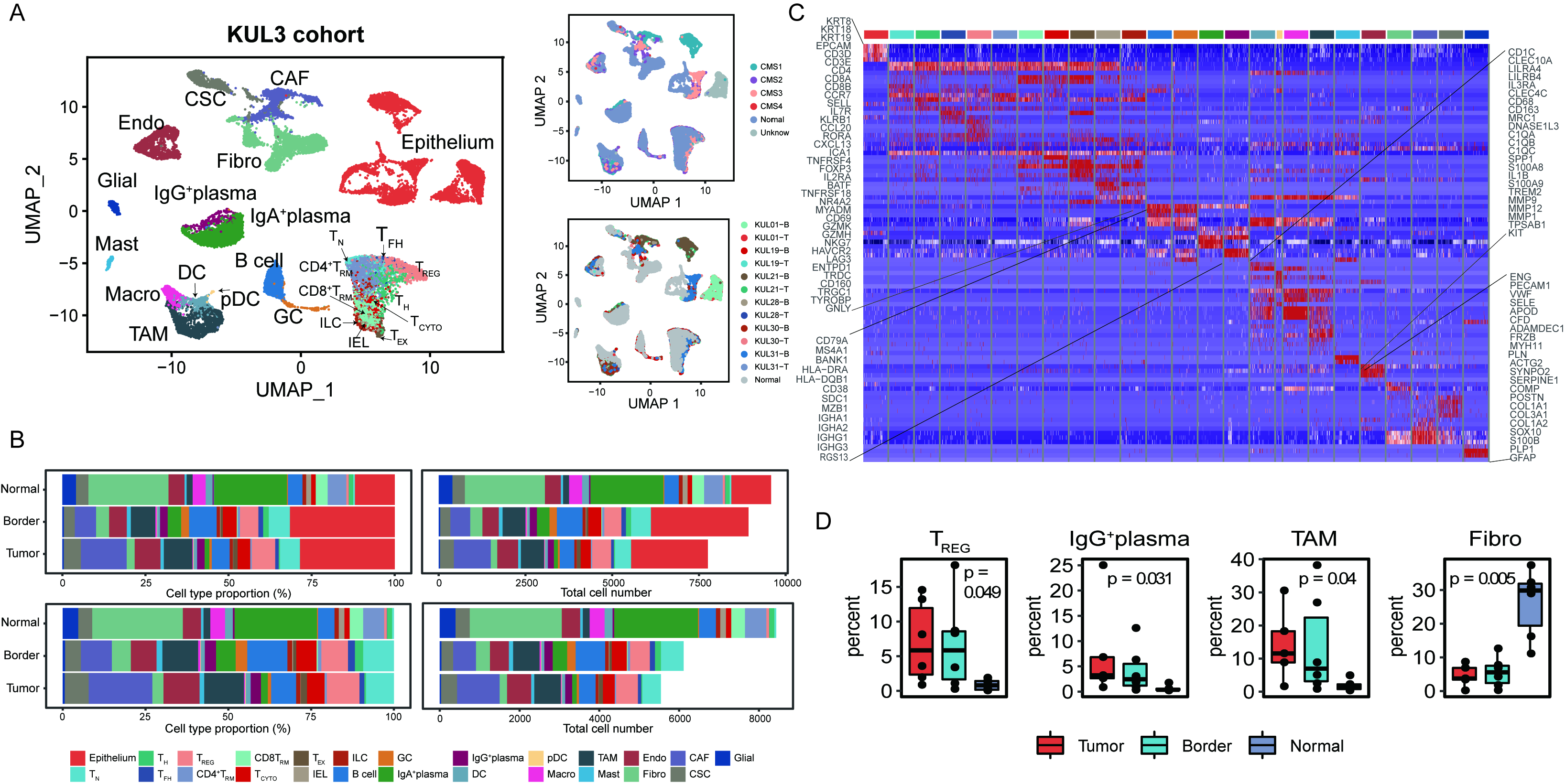

Supplement: Supplementary file 8 — Additional file 8: Figure S1. Cellular landscape of CRC in the KUL3 cohort. A. UMAP plot of 26,268 cells colored by cell cluster, CMS and sample origin. Each dot represents a cell and cellular cluster is annotated with text. B. Proportions of the identified cell clusters distributed across tumor, border and adjacent normal tissues with the relative cell type proportions and total cell numbers. Upper: all cell clusters; lower: immune and stromal cell clusters. C. Heatmap of representative markers for the cell clusters. A total of 100 random cells in each cluster were chosen for visualization. The color legend is as in B. D. Frequencies of the selected cell types for tumor, border and adjacent normal samples. The Kruskal-Wallis test p value is shown. [file 12967_2022_3661_MOESM8_ESM.tif]

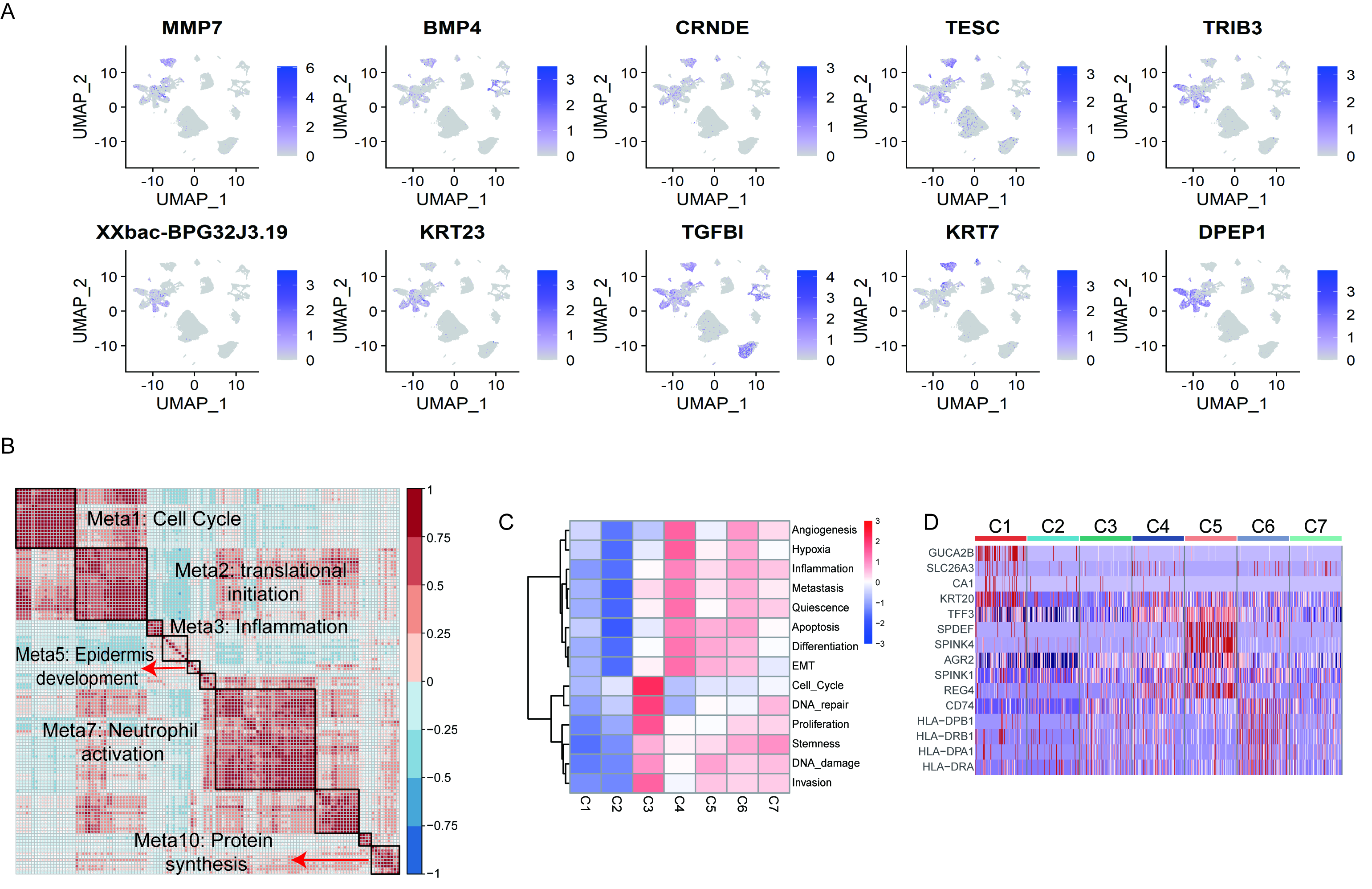

Supplement: Supplementary file 9 — Additional file 9: Figure S2. Functional heterogeneity of CRC epithelial cells in the SMC cohort. A. UMAP feature plot of the top 10 upregulated genes in the SMC cohort. B. Correlation heat map of 123 programs identified by cNMF. The Pearson correlation coefficient is indicated by the color. C. Heatmap of the relative mean signature score of cancer cell functional signatures across C1-C7. The signature score was calculated using the “AUCell” package. D. Heatmap of functional genes across C1-C7. A total of 100 random cells in each cluster were used for visualization [file 12967_2022_3661_MOESM9_ESM.tif]

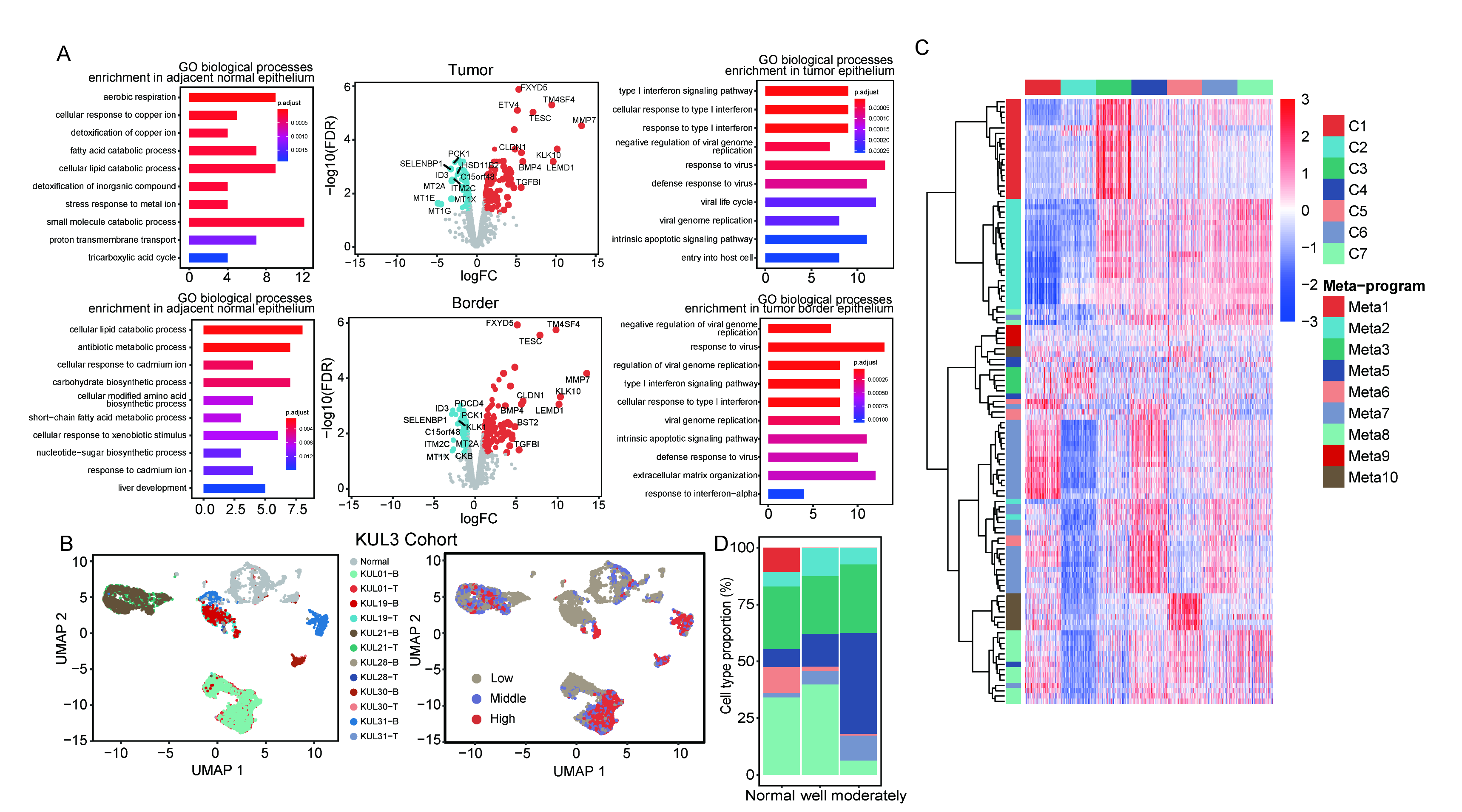

Supplement: Supplementary file 10 — Additional file 10: Figure S3. Epithelial heterogeneity validation in KUL3 cohort. A. Volcano plot of the differentially expressed genes between colorectal cancer cells at the core (up) or border (down) and adjacent normal epithelial cells. Genes with FDR values less than 0.05 and absolute log2FC values greater than 1 are colored blue (upregulated in adjacent normal epithelial cells) or red (upregulated in cancer and border tissues). GO enrichment plot is shown on each side with the bar color indicating enrichment significance, and bar length showing the number of overlapping dysregulated genes and the GO term. B. UMAP plot of 6,178 epithelial cells from 6 patients colored by patient and tissue type (tumor, border and normal). C. Hierarchical heat map of 115 expression programs. The cells in each cluster were down-sampled to 100. The corresponding meta-programs are listed in the rows. D. Relative proportions of epithelial cell clusters for adjacent normal tissues and colorectal adenocarcinoma samples of different histological grades [file 12967_2022_3661_MOESM10_ESM.tif]

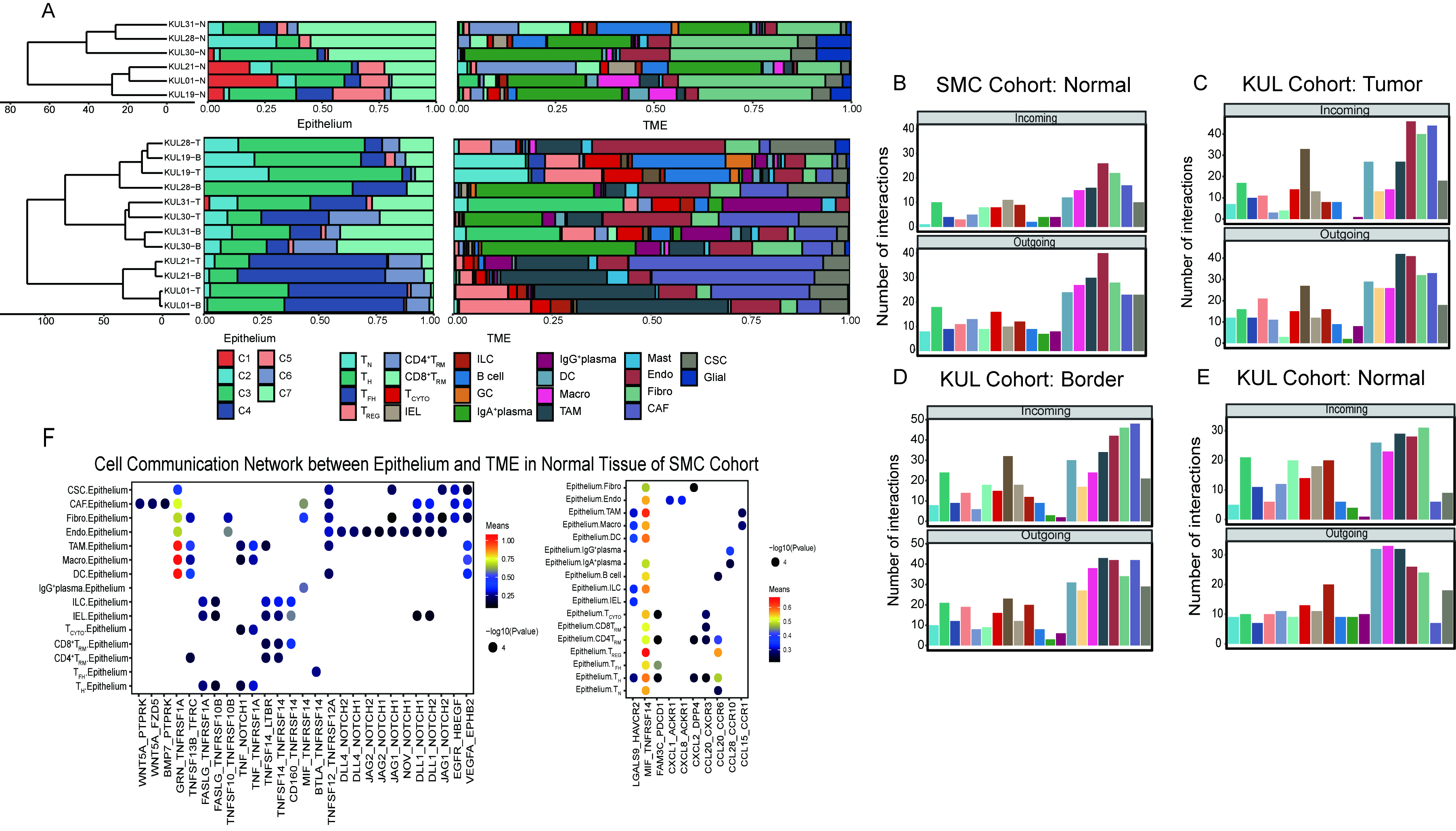

Supplement: Supplementary file 11 — Additional file 11: Figure S4. Dynamic interactions between the EC and MC. A. Relative proportions of epithelial cell clusters and TME cell clusters in the KUL3 cohort. Samples were clustered according to the distribution pattern of epithelial cells with the corresponding TME cell cluster distribution shown in the right panel. B–E. Bar plot showing the number of incoming events and outgoing events for epithelial cells communicating with TME cells in normal tissues of the SMC cohort (B), tumor tissues of the KUL3 cohort (C), border tissues of the KUL3 cohort (D) and normal tissues of the KUL3 cohort (E). F. Bubble plots of ligand-receptor pairs between epithelial cells and TME cells in normal tissues of the SMC cohort. Dot size and color represent the enrichment scores and the relative mean expression level of ligand-receptor pairs, respectively. [file 12967_2022_3661_MOESM11_ESM.tif]

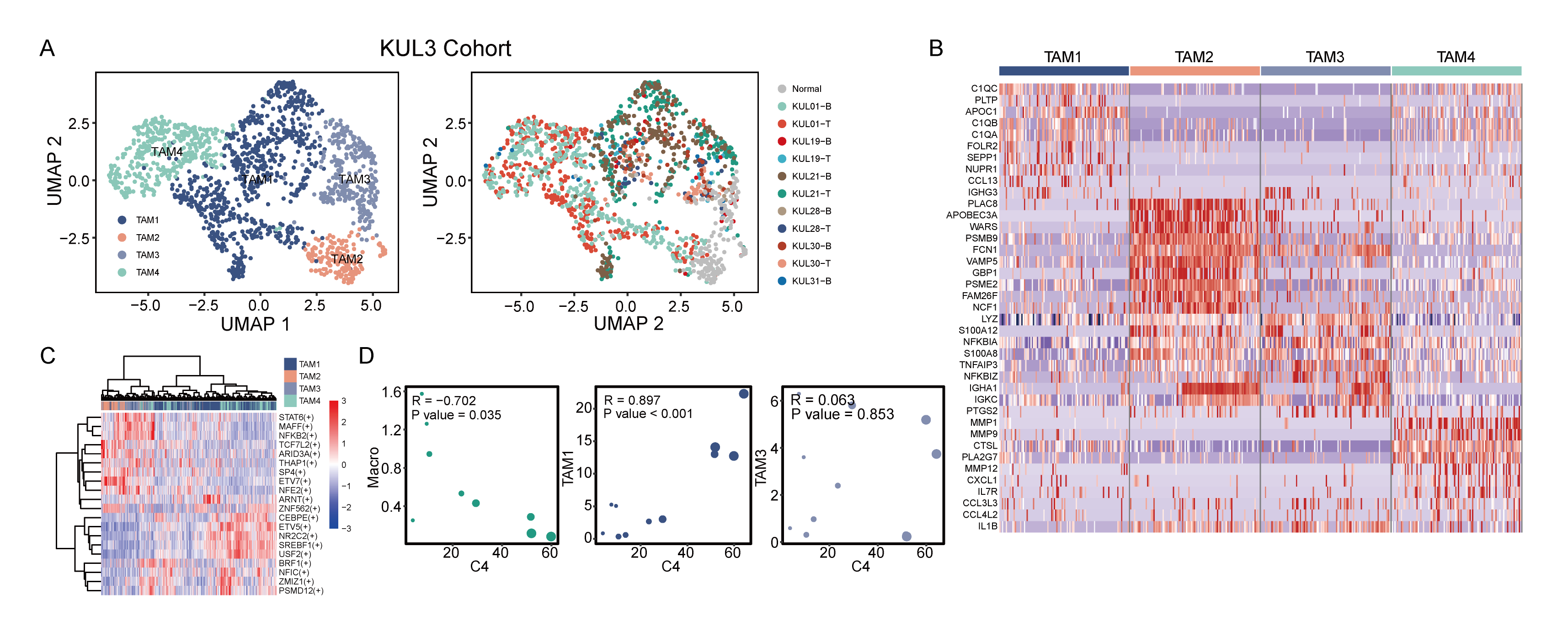

Supplement: Supplementary file 12 — Additional file 12: Figure S5. Characterization of myeloid cells in the KUL3 cohort. A. UMAP plot of myeloid cells colored by cell cluster and sample origin. B. Heatmap of marker genes identified through Seurat. For each cell cluster, cells were down-sampled to 100. C. Heatmap plot of the top 5 ranked regulons in TAM1-TAM4 identified by pySCENIC. D. Dot plot of the correlation between the proportions of C4 cells in epithelial cells and macrophages (left), TAM1 (middle) and TAM3 (right) in the TME. Each dot represents a patient, and a larger dot size means a higher C4 cell proportion. [file 12967_2022_3661_MOESM12_ESM.tif]

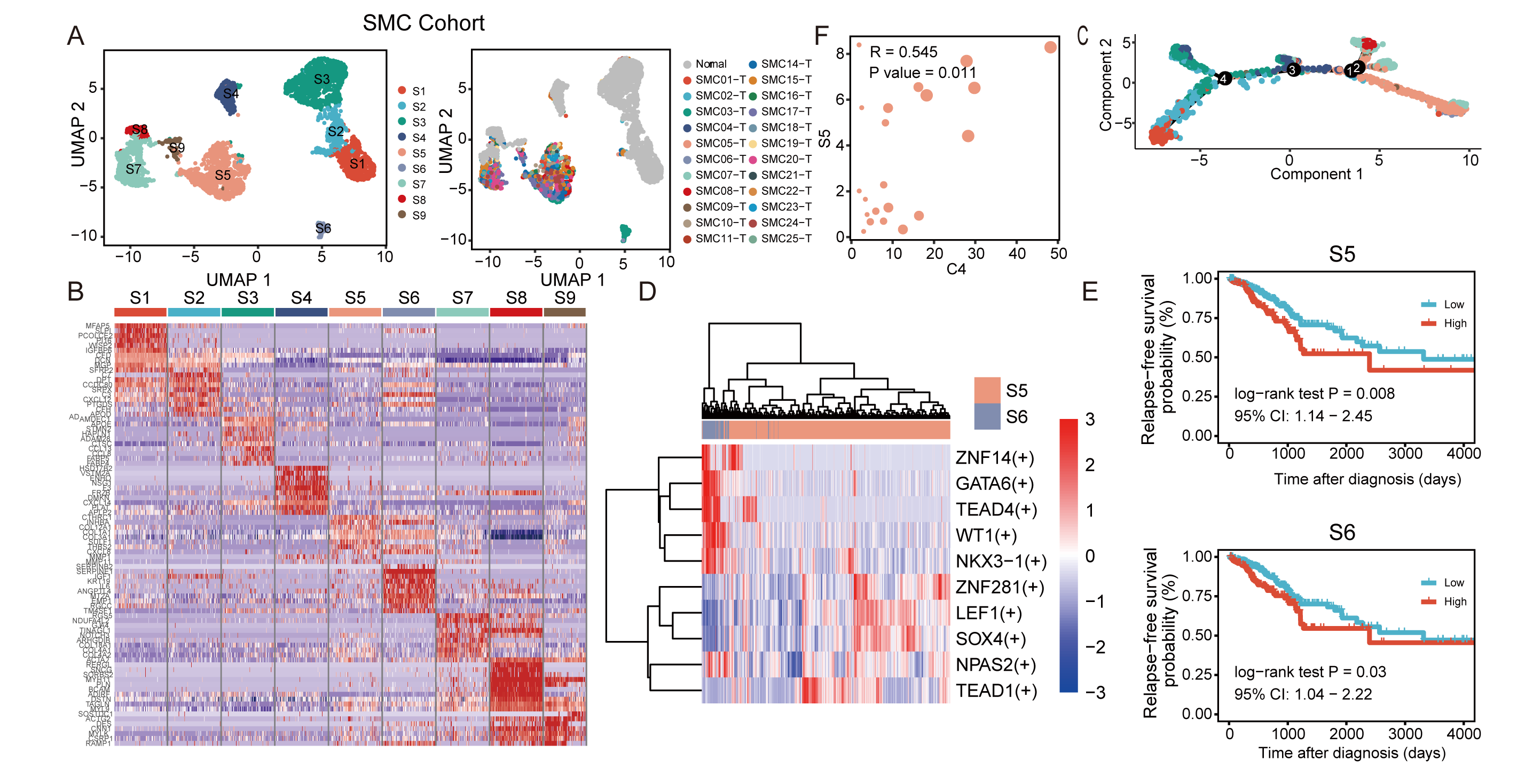

Supplement: Supplementary file 13 — Additional file 13: Figure S6. Characterization of stromal cells in the SMC cohort. A. UMAP plot of stromal cells colored by cell cluster and sample origin. B. Heat map of marker genes identified through Seurat. For each cell cluster, cells were down-sampled to 100. C. Differentiation trajectories inferred by Monocle2. Dots represent stromal cells and are colored by identified cell cluster. D. Heat map of the top 5 ranked regulons in S5 and S6. E. Relapse-free survival curves for S5 (top) and S6 (bottom) in the TCGA-COADREAD cohort. F. Dot plot of the correlation between the proportions of C4 cells in epithelial cells and S5 in the TME. Each dot represents a patient, and a larger size means a higher C4 cell proportion. Correlation test is estimated by Pearson correlation test. [file 12967_2022_3661_MOESM13_ESM.tif]

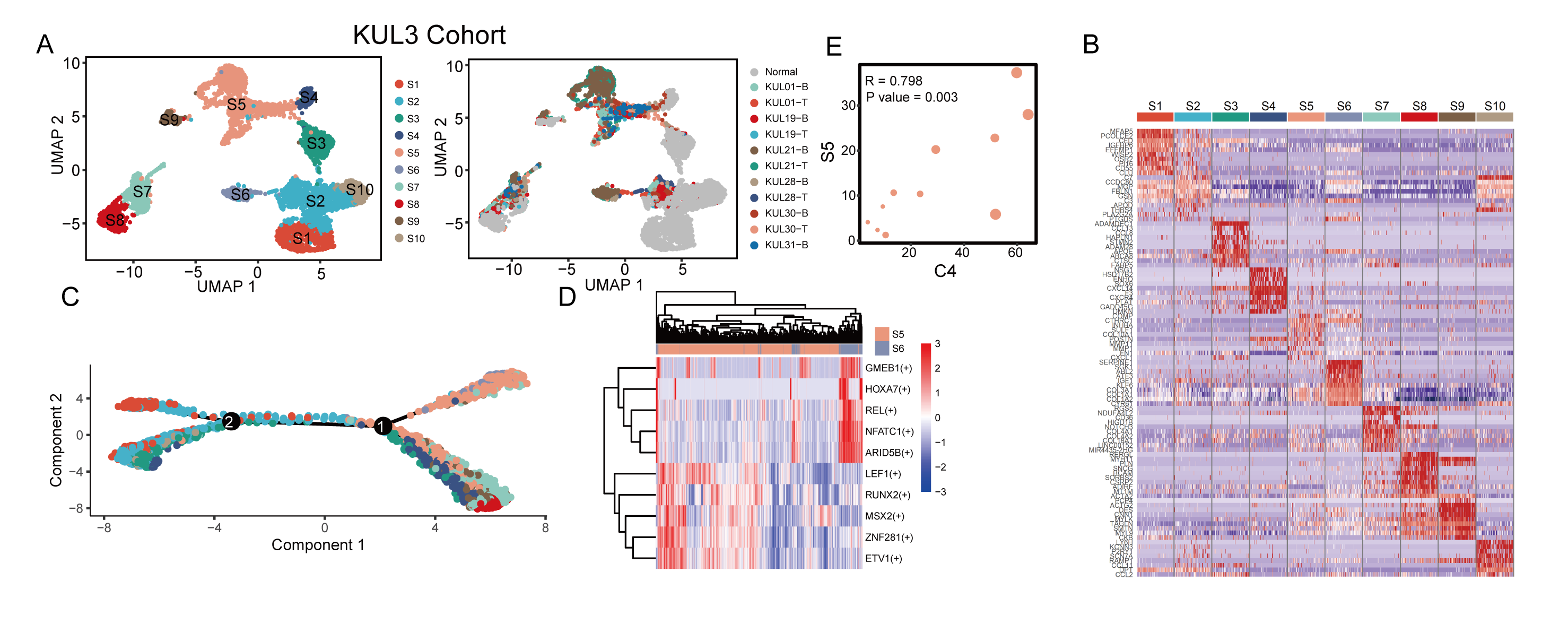

Supplement: Supplementary file 14 — Additional file 14: Figure S7. Characterization of stromal cells in the KUL3 cohort. A. UMAP plot of stromal cells colored by cell cluster and sample origin. B. Heat map of marker genes identified through Seurat. For each cell cluster, cells were down-sampled to 100. C. Differentiation trajectory inferred by Monocle2. Dots represent stromal cells colored by identified cell cluster. D. Heat map plot of the top ranked regulons. E. Dot plot of the correlation between the proportions of C4 cells in epithelial cells and S5 cells in the TME. Each dot represents a patient, and a larger size means a higher C4 cell proportion. Correlation test is estimated by Pearson correlation test. [file 12967_2022_3661_MOESM14_ESM.tif]

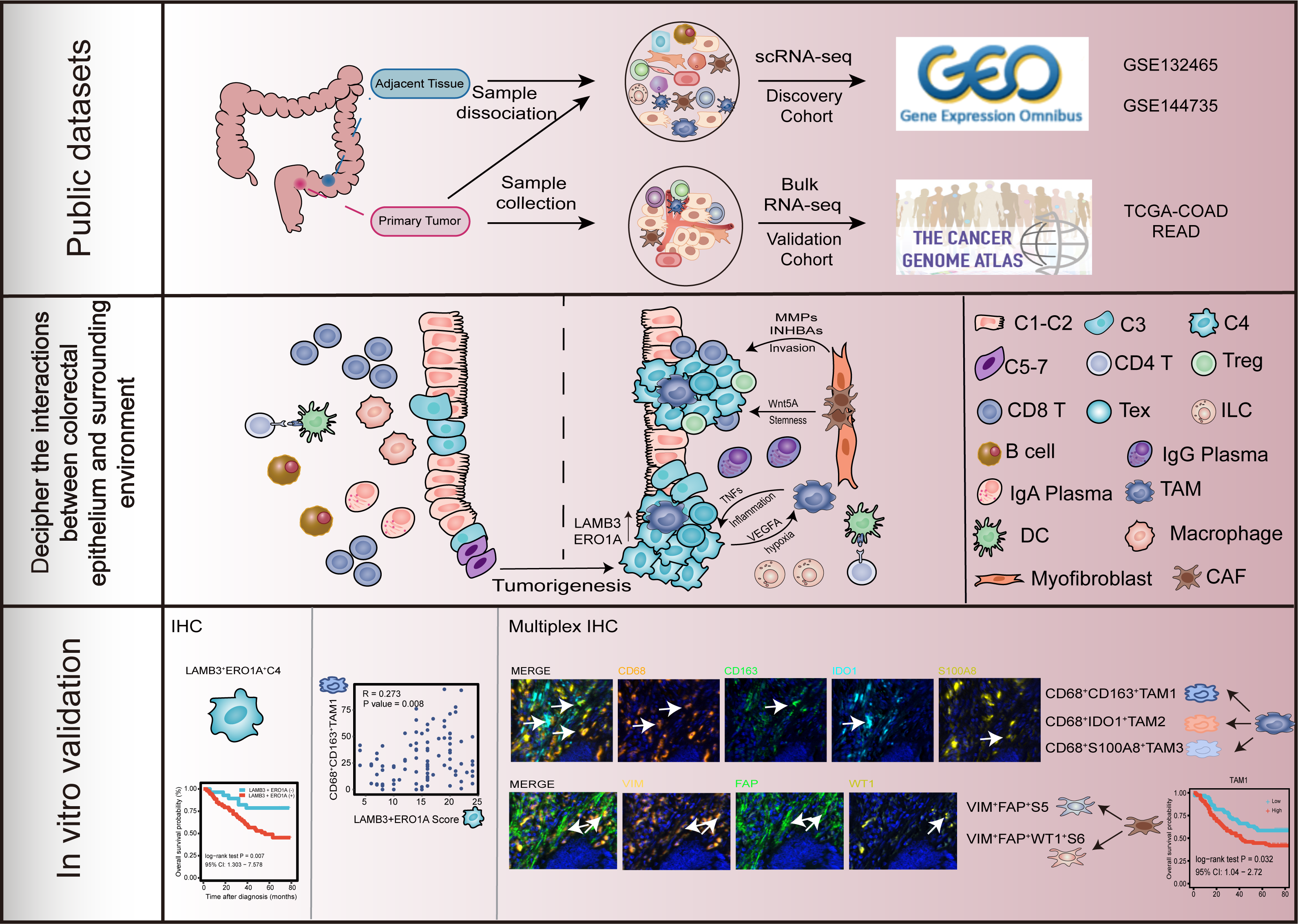

Supplement: Supplementary file 15 — Additional file 15: Figure S8. Workflow of this study. Single-cell transcriptomes and bulk RNA-seq data were integrated to fully analyze the complicated relationship between colorectal epithelium and surrounding environment. C4 cells were featured with high invasive potential and related with TAMs and CAFs, and further validated in vitro using IHC and mIHC. [file 12967_2022_3661_MOESM15_ESM.tif]
